# Exploring milk loss and variability during environmental perturbations across lactation stages as resilience indicators in Holstein cattle

**DOI:** 10.3389/fgene.2022.1031557

**Published:** 2022-12-02

**Authors:** Ao Wang, Luiz F. Brito, Hailiang Zhang, Rui Shi, Lei Zhu, Dengke Liu, Gang Guo, Yachun Wang

**Affiliations:** ^1^ Key Laboratory of Animal Genetics, Breeding and Reproduction, Ministry of Agriculture of China, National Engineering Laboratory for Animal Breeding, College of Animal Science and Technology, China Agricultural University, Beijing, China; ^2^ Department of Animal Sciences, Purdue University, West Lafayette, IN, United States; ^3^ Hebei Sunlon Modern Agricultural Technology Co., Ltd., Dingzhou, China; ^4^ Beijing Sunlon Livestock Development Co., Ltd., Beijing, China

**Keywords:** milk variability, daily milk yield, cattle resilience, lactation curve, dairy cattle

## Abstract

Genetic selection for resilience is essential to improve the long-term sustainability of the dairy cattle industry, especially the ability of cows to maintain their level of production when exposed to environmental disturbances. Recording of daily milk yield provides an opportunity to develop resilience indicators based on milk losses and fluctuations in daily milk yield caused by environmental disturbances. In this context, our study aimed to explore milk loss traits and measures of variability in daily milk yield, including log-transformed standard deviation of milk deviations (Lnsd), lag-1 autocorrelation (Ra), and skewness of the deviations (Ske), as indicators of general resilience in dairy cows. The unperturbed dynamics of milk yield as well as milk loss were predicted using an iterative procedure of lactation curve modeling. Milk fluctuations were defined as a period of at least 10 successive days of negative deviations in which milk yield dropped at least once below 90% of the expected values. Genetic parameters of these indicators and their genetic correlation with economically important traits were estimated using single-trait and bivariate animal models and 8,935 lactations (after quality control) from 6,816 Chinese Holstein cows. In general, cows experienced an average of 3.73 environmental disturbances with a milk loss of 267 kg of milk per lactation. Each fluctuation lasted for 19.80 ± 11.46 days. Milk loss traits are heritable with heritability estimates ranging from 0.004 to 0.061. The heritabilities differed between Lnsd (0.135–0.250), Ra (0.008–0.058), and Ske (0.001–0.075), with the highest heritability estimate of 0.250 ± 0.020 for Lnsd when removing the first and last 10 days in milk in a lactation (Lnsd2). Based on moderate to high genetic correlations, lower Lnsd2 is associated with less milk losses, better reproductive performance, and lower disease incidence. These findings indicate that among the variables evaluated, Lnsd2 is the most promising indicator for breeding for improved resilience in Holstein cattle.

## 1 Introduction

Dairy cows are affected by many environmental disturbances throughout their lives (Friggens et al., 2017; Berghof et al., 2018; Silpa et al., 2021), including diseases (Rajala-Schultz et al., 1999a), heat stress (Polsky and von Keyserlingk, 2017; Shi et al., 2021; Wankar et al., 2021), cold stress (Hu et al., 2021a), reproductive events (Macciotta et al., 2011; Guarini et al., 2019), and feed availability and quality (Friggens et al., 2016). These disturbances often result in temporary drop or continuous fluctuations in daily milk yield, which can be considered as milk losses relative to the expected lactation curve (Ben et al., 2021). The pattern of milk losses differs among cows and events and can last for long periods. For instance, mastitis events could affect milk yield for more than 30 days, with a milk loss of 50–300 kg per event (van Soest et al., 2016; Adriaens et al., 2021a). Milk fever can result in lower milk yield for up to 6 weeks with milk losses ranging from 1.1 to 2.9 kg per day (Rajala-Schultz et al., 1999b). Intensive genetic selection for milk production traits has led high-yielding cows to experience negative energy balance (NEB) more often in early lactation, which in turn can result in higher incidences of metabolic disorders (Friggens et al., 2013; Brito et al., 2021). Heat stress also contributes to a reduction in milk yield by affecting endocrine and metabolism processes (Wankar et al., 2021), with reports of milk yield declining by approximately 0.41 kg/d when the temperature and humidity index (THI) exceeds 69 (Bouraoui et al., 2002). However, in the past, production performance and lactation dynamics were mainly analyzed using low frequency test-day records (e.g., weekly or monthly; Adriaens et al., 2021b) due to limitations in large-scale data recording. Disturbances are difficult to be monitored when they are of short duration and in the middle of a test-day interval (Elgersma et al., 2018). With the spread of high frequency milk recording equipment, longitudinal data generated by sensors may contain additional information for deriving novel breeding goals (Peng et al., 2009; Brito et al., 2020, 2021).

To study perturbations in milk production, a theoretically undisturbed lactation curve–the expected lactation curve (ELC), needs to be predicted. The overall objective of predicting an ELC is to eliminate the effect of short-term environmental perturbations on daily milk yield and to reduce the variability, thus enabling the characterization of the lactation potential of each cow in the absence of environmental perturbations (Ben et al., 2021). Identifying environmental perturbations to fit ELC is difficult as information about disturbances is often unavailable (Garcia-Baccino et al., 2021). Therefore, it becomes a mainstream approach to calculate ELC from the actual daily milk yield. Compartment model (Ben et al., 2021), fourth-order polynomial quantile regression model (Poppe et al., 2020), nonparametric trend model (Poppe et al., 2020), and Wood model incorporating iterative procedures (Adriaens et al., 2021a; 2021b) have been used to fit ELC. An important limitation of these approaches is the generalization of the ELC to a single model, thus ignoring differences in lactation trends among cows, which is a topic interest of this current study.

The deviation between the observed and expected daily milk yield can be used for describing the longitudinal dynamics of milk yield and identifying milk losses (Adriaens et al., 2021b). Describing deviations in daily milk yield is needed for evaluating the impact of environmental disturbances in milk yield and for applying effective management decisions. Meanwhile, this provides an opportunity for studying the resilience of lactating cows. Resilience can be defined as the animals’ ability to maintain their level of production under environmental disturbances or to recover rapidly to the state pertained before exposure to an environmental disturbance (Colditz and Hine, 2016). Resilience has not been included in any national dairy cattle selection goal to date (Berghof et al., 2018; Poppe et al., 2022b). This is due to the insufficient research on the definition of the best approaches for quantifying resilience, biological validation of resilience indicators, and the selection directions for resilience which are partially encompassed by health, reproduction, and longevity traits in the current selection goals. Genetically selecting for improved resilience could improve herd productivity (Colditz and Hine, 2016; Poppe et al., 2022b), result in better animal welfare (Mulder and Rashidi, 2017), reduce the use of drugs and antibiotics for treating diseases (Konig and May, 2019), and is significantly associated with easier management and lower production cost of herds (Berghof et al., 2018). Many studies have proposed a data-driven approach to derive resilience indicators based on longitudinal data such as daily milk yield (Elgersma et al., 2018; Poppe et al., 2020; Adriaens et al., 2021b; Ben et al., 2021). These methods rely on the assumption that individuals with less fluctuation in longitudinal records are more resilient than those with greater variability. Poppe et al. (2020) used fluctuations in daily milk yield to derive resilience indicators and proposed the log-transformed variance of deviations from lactation curves as the best indicator. Elgersma et al. (2018) defined three traits related to the number of drops in milk yield using the Student *t* test and found that the variance of milk production is the best resilience indicator to predict udder health, ketosis, and longevity. Optimal resilience indicators should have high heritability to enable effective genetic selection for practical applications and ideally favorable genetic correlation with economically important traits. However, the potential of milk loss traits, which directly reflect fluctuations in daily milk yield (e.g., magnitude and duration of milk loss), as suitable resilience indicators has not been previously explored. Furthermore, although resilience indicators based on variability in longitudinal data have been proposed, the calculation of resilience indicators and the genetic relationships with traits already included in selection indexes need to be explored in Chinese Holstein herds.

In this context, the main objectives of this study were 1) to characterize lactation curves and milk yield variability in Holstein cattle; and 2) to investigate the genetic background of milk loss traits and variability traits as resilience indicators and their genetic correlations with economically important traits. Results of this study will contribute to the identification of appropriate resilience indicators to be used for genetically improving resilience of high-yielding Holstein cattle.

## 2 Materials and methods

### 2.1 Datasets

A total of 11,536,488 daily milk yield records from 22,666 Holstein cows raised in three herds (owned by a single entity) located in Hebei (China) were available for this study. The data was collected from January 2017 to January 2021. The daily milk yield of each cow was extracted from the farm management software. Animals were housed in free-stall systems, fed total mixed rations, and milked three times per day on rotary milking systems. The pedigree of cows with phenotypic records after data editing were traced back as many generations as possible. The final pedigree included 21,574 females and 2,447 males born from 1907 to 2018.

Additional economically important traits were also included in this study. Five reproduction traits were evaluated, including age at first calving in heifers (AFC), age at first insemination in heifers (AFS), interval from first to last insemination in heifers (IFL_H) and cows (IFL_C), and interval from calving to first insemination (ICF), all measured in days. Additional details about the definition of the reproduction traits can be found in Guo et al. (2014) and Liu et al. (2017). Three longevity traits, also measured in days, included the number of days from the first calving to the end of the first (Lon1) and second (Lon2) lactation or culling, and productive life (PL), which refers to the number of days from the first calving to culling or death. The definitions of the longevity traits are described in Zhang et al. (2021). Furthermore, four health traits included udder health (UDDE), reproductive disorders (REPR), metabolic disorders (METB), and digestive disorders (DIGS), as detailed in Wang et al. (2022). The health traits were defined as binary traits with a value of one indicating if a cow had at least one health problem at any time during the corresponding lactation, and 0 otherwise. The number of individuals with reproduction traits, longevity traits, and health traits ranged from 3,871 (IFL_H) to 8,860 (ICF), 883 (PL) to 2,610 (Lon1), and 5,921 (METB and DIGS) to 7,347 (UDDE and REPR), respectively. These traits were recorded until June 2021. The descriptive statistics of these traits used to estimate genetic correlations are presented in Supplemental Table S1.

### 2.2 Data analyses

#### 2.2.1 Data pre-processing

From the initial dataset, only milk yield records measured from days in milk (DIM) 1–305 days, milk yield from 2.5 to 100 kg per day, and non-duplicated records were retained for further analyses. Only cows with age at first calving between 600 and 1,800 days were included in the study. The specific data editing steps, with information on the quality control used, the number of cows, lactations, and records after each editing step, are presented in Supplemental Table S2 (Items 1–9). After the quality control, 22,366 lactations (parity 1 = 7,995; parity 2 = 6,160; parity 3 + = 8,211) were kept for further analyses. A total of 27.61% of lactations had more than 300 milk yield records and 1.85% of the lactations had all 305 milk yield records.

#### 2.2.2 Lactation clustering

Cluster analysis was performed on all lactations in order to group lactations with similar patterns of daily milk yield. The objectives of clustering were 1) to identify and eliminate outliers in each group, 2) to obtain the expected milk yield for missing values for DIM 1–4 days and DIM 305 within the imputation process of missing daily records described in Section 2.2.3, and 3) to account for differences in lactation patterns in the statistical models fitted for resilience indicators.

To minimize clustering divergences caused by differences in the range of milk yield per lactation and emphasize inter-cluster homogeneity (Lee et al., 2020), the phenotypic records were normalized based on the Z-score transformation method. Afterwards, for DIM 31 to 270 within each lactation, the average milk yield for each 10 days was calculated, and 24 average values for each lactation per cow were obtained. Based on these average milk yield records, a principal component analysis (PCA) was performed, and the first five principal components (PC1 to PC5) accounting for 70% of the total variation were considered as attribute points to further measure the similarity across lactations.

The Agglomerative Hierarchical Clustering algorithm (Murtagh and Contreras, 2011) was used to cluster and group lactations, and Euclidean distance was used to measure intra-class distances between two lactations as (Warren Liao, 2005):

$$d\left(A,\;B\right)=\sqrt{\overset{N}{\underset{i=1}{\sum }}{\left(m_{A,i}-m_{B,i}\right)}^{2}}$$

where d(A, B) is the distance between lactations A and B; $m_{A,i}$ and $m_{B,i}$ are the i^th^ PC in lactation A and B, respectively; and, N is the total number of PCs (equal to 5). To minimize the square sum of intra-class deviations and maximize the square sum of inter-class deviations, the Ward linkage method was used to measure inter-class distance between group pairs (Murtagh and Contreras, 2011).

**FIGURE 1:**
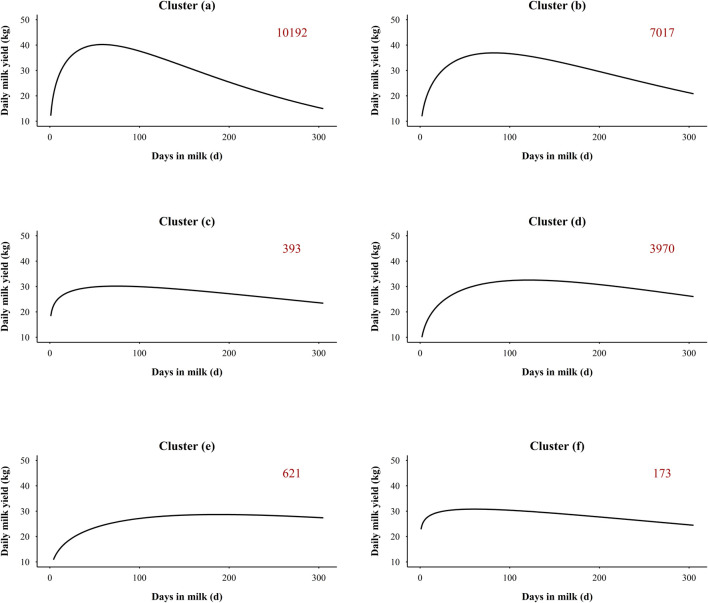
Average daily milk yield in six lactation clustering groups. The number in the upper right corner indicates the number of lactations in each cluster. **(A–F)** refer to cluster group (a), (b), (c), (d), (e), and (f), respectively.

The silhouette coefficient was adopted for the selection of the number of clusters (Aranganayagi and Thangavel, 2007). Six was the most appropriate number of clusters due to the highest silhouette coefficient, and additional details about the silhouette coefficient of different number of clusters were presented in Supplemental Table S3. The average trends of daily milk yield for each cluster are presented in Figure 1. Among the six groups, the largest cluster (Figure 1A) included 10,192 lactations (45.57%), while only 173 lactations (0.77%) were included in the smallest cluster (Figure 1F). Descriptive statistics on lactation clustering of the final dataset are detailed in Section 3.1. Within each group, the records deviating three or more SD from the mean were removed for each DIM. A total of 96,601 outlier records (1.50%) were removed as detailed in Supplemental Table S2 (Item 10).

#### 2.2.3 Phenotypic data imputation

To obtain complete daily milk yield records from DIM 1 to 305, the missing records were imputed for each lactation. For missing values for DIM 1–4 days and DIM 305, the normalized average milk yield of the corresponding DIM in each cluster was used. Missing milk yield was calculated as the normalized value multiplied by the standard deviation of the non-missing milk yield of the lactation and then added to the mean. After a series of quality control on the record distribution, there was little difference between the average milk yield calculated *via* non-missing values and the true average milk yield. For DIM five to 304, the missing records were sequentially imputed using linear regression interpolation in order of DIM. A total of five records from days n − 4, n − 3, n − 2, n − 1, and n + k was used to fit a first-order linear regression model, where k was the number of days between day n and the next day where daily milk yield was recorded. The regression value for day n was the filled value on that day until all missing values were filled in for each lactation. After imputation, 305 records of daily milk yield for 22,366 lactations were obtained as detailed in Supplemental Table S2 (Item 11).

### 2.3 Fitting individual lactation curves

**FIGURE 2:**
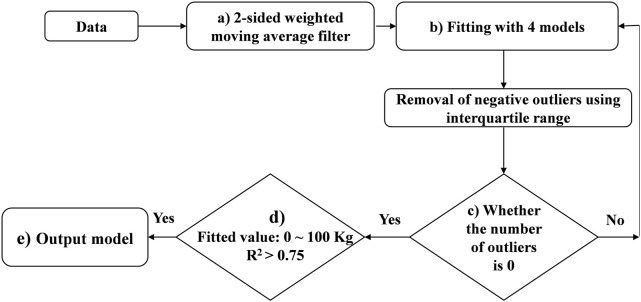
Illustrative scheme of the process of fitting the expected lactation curve (ELC).

To obtain the expected lactation curve (ELC) of each parity, an iterative procedure was implemented for each lactation with the method presented in Figure 2, and the detailed steps are as follows:

1) A 2-sided weighted moving average filter with a window of 5 days was established in process (a), which means that the expected milk yield on a certain day ( $x_{t}$ ) is the weighted average of the milk yield in day $x_{t-2}$ , $x_{t-1}$ , $x_{t}$ , $x_{t+1}$ , and $x_{t+2}$ . The formula is as follows:

$$x_{t}=0.1x_{t-2}+0.2x_{t-1}+0.4x_{t}+0.2x_{t+1}+0.1x_{t+2}$$

2) In the first iteration, it was assumed that the expected shape of the optimal lactation curve for each lactation was different. In process (b), four lactation curve models were used to fit each lactation on all data, including the Wood (Wood, 1967), Nelder (Nelder, 1966), Wilmink (Wilmink, 1987), and Ali-Schaeffer (Ali and Schaeffer, 1987) models. The four models can be described as:

$$Y_{t}=at^{b}e^{-ct}\left(Wood\;model\right)$$

$$Y_{t}^{-1}=a+bt^{-1}+ct\left(Nelder\;model\right)$$

$$Y_{t}=a+bt^{-0.05t}+ct\left(Wilmink\;model\right)$$

$$Y_{t}=a+bt+ct^{2}+d\log t+e{\left(\log t\right)}^{2}\left(Ali-Schaeffer\;model\right)$$

Where $Y_{t}$ is the daily milk yield, t is DIM and a, b, c, d, and e are the model parameters.

3) Calculate determination coefficient (R^2^) of the four models and select the model with the highest R^2^ as the optimal model for that lactation for the subsequent iterative procedure.
4) Calculate the deviations between the actual values and the fitted values for each DIM currently retained (for the first iteration, the number of deviations is 305), as well as the lower quartile (LQ) and the interquartile ranges (IQR) of these deviations.
5) Remove all data with deviation less than LQ-1.5*IQR as outliers to obtain the filtered data resulting from the iteration.
6) Check whether the number of outliers is 0 (as process (c) showed). If not, fit the same lactation curve model on the filtered data from the previous step, and calculate the R^2^ of the model.
7) Repeat steps (4) to (6) until no outliers are identified. Up to this step, we obtained ELC for each lactation.
8) In process (d), a secondary quality control for ELC was performed. Only ELC with daily milk yield between 0 and 100 kg and R^2^ (based on the last iteration) > 0.75 were kept in this study.

Furthermore, the lactations with 305 days milk yield deviating three or more SD from the mean and cows with unknown parents were excluded. Finally, 8,935 lactations were obtained for 6,816 cows, as detailed in Supplemental Table S2 (Items 12–14).

### 2.4 Definition of milk loss traits and variability traits as resilience indicators

**FIGURE 3:**
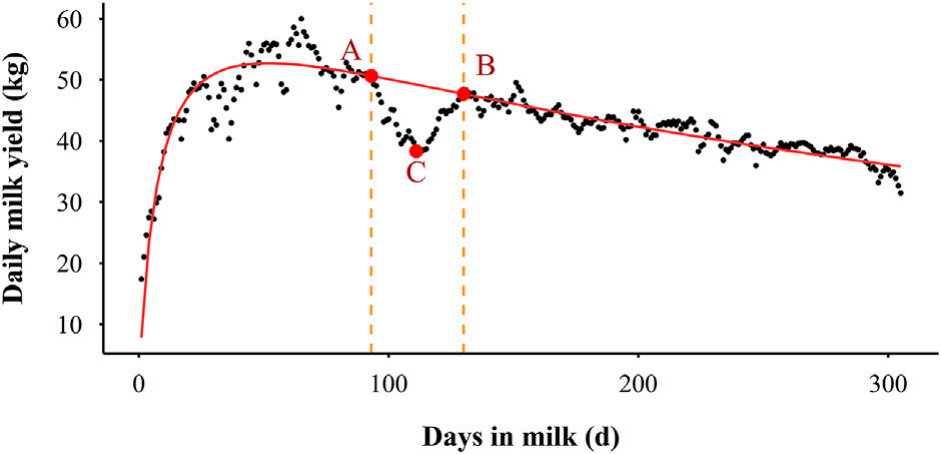
An illustrative example of the definition of the milk fluctuation phase. The scatter indicates the actual daily milk yield, the red line represents the expected lactation curve (ELC), the section AB is a fluctuation phase, point A is the start of the fluctuation, point B is the end of the fluctuation, and point C is the highest decrease of the fluctuation.

In this study, the deviations between actual records and the ELC fitted values for each lactation were calculated and expected to contain information about resilience and response to environmental disturbances in Holstein cows. These deviations were expected to be around zero in the absence of perturbations, while during perturbations they would be consistently negative. The number of deviations was 305 for a lactation. A fluctuation was defined as a period of at least 10 successive days of negative deviations for which the milk yield dropped at least once below 90% of the ELC fitted values. An example to illustrate the definition is presented in Figure 3, where the scatters are the daily milk yield in a lactation and the red line indicates the ELC. The section AB is a fluctuation phase. The DIM at points A and B are the beginning and ending of this fluctuation, and the DIM at point C is the highest decrease of this fluctuation. Based on the definitions of deviation and fluctuation, two types of traits were considered as potential resilience indicators in this study: milk loss traits which directly reflect fluctuations in daily milk yield and variability traits obtained by the deviations.

The 305 days milk yield (MY305) and milk loss traits such as the milk loss (ML; in Kg), the number of ML events (NML), the total duration of ML events within a lactation (TDML; in days), the percentage of ML to MY305 (MLP; in %), the duration of each ML period (DML; in days), and milk loss in each ML period (MLF; in Kg) were calculated for each parity. MY305 is calculated by summing up the imputed daily milk yield which included both measured and imputed daily records. ML refers to the sum of the daily milk yield which dropped in all fluctuation phases in a lactation. NML refers to the number of fluctuation events for daily milk yield per lactation (i.e., number of ML). TDML refers to the total duration (in days) of all fluctuation per lactation. MLP refers to the proportion of ML to MY305 per lactation. DML and MLF refer to the duration (in days) and ML in each fluctuation per lactation, respectively. Thus, there may be more than one DML and MLF per lactation.

Through the definitions of deviation, three variability traits were explored within each parity: log-transformed standard deviation of milk deviations (Lnsd), lag-1 autocorrelation of milk deviations (Ra), and skewness of milk deviations (Ske). To identify the effect of lactation stage on resilience, these three variability traits were calculated based on four periods: the entire lactation (Lnsd1, Ra1, and Ske1, from DIM 1–305), lactation period when removing the first and last 10 days (Lnsd2, Ra2, and Ske2, from DIM 11–295), during the lactation peak period (Lnsd3, Ra3, and Ske3, from DIM 60–90), and the period consisting of each DIM when the actual milk yield was below the ELC fitted value (Lnsd4, Ra4, and Ske4).

### 2.5 Genetic analyses

#### 2.5.1 Estimation of genetic parameters

The GLM procedure of the SAS software (version 9.4; SAS Institute Inc.) was performed to identify the systematic effects that should be included in the genetic models on milk loss traits and variability traits. Variance and co-variance components were estimated using the Average Information Restricted Maximum Likelihood algorithm implemented in the DMU software (Madsen et al., 2006). Heritability of MY305, milk loss traits (ML, NML, TDML, and MLP), and all variability traits was estimated based on single-trait animal model and heritability of DML and MLF was estimated based on single-trait repeatability animal model.

The single-trait animal model used can be described as:

$$\begin{array}{c} y_{ijklmnp}={hys}_{i}+p_{j}+c_{k}+m_{l}+{afc}_{m}+a_{n}+e_{ijklmnp} \end{array}$$ (1)

where $y_{ijklmnp}$ are the phenotypic records for MY305, milk loss traits (ML, NML, TDML, and MLP), and all variability traits, ${hys}_{i}$ is the fixed effect of herd-calving year-calving season (42 levels); $p_{j}$ is the fixed effect of parity (five levels, including 1, 2, 3, 4, and 5+); $c_{k}$ is the fixed effect of cluster group (six levels); $m_{l}$ is the fixed effect of lactation curve model (four levels–the four lactation models described in Section 2.3); ${afc}_{m}$ is the fixed effect of age at first calving (four levels, including 22 or less months of age, 23 to 24, 25 to 26, and 27 months and older); $a_{n}$ is the random additive genetic effect; $e_{ijklmnp}$ is the random residual effect. It was assumed that $a\;∼\;N\left(0,A\sigma_{a}^{2}\right)$ and $e\;∼\;N\left(0,\;I\sigma_{e}^{2}\right)$ , where A is the matrix of additive genetic relationships constructed based on pedigree information, $\sigma_{a}^{2}$ is the additive genetic variance, I is an identity matrix, and $\sigma_{e}^{2}$ is the residual variance.

The single-trait repeatability animal model can be described as:

$$\begin{array}{c} y_{ijklmnpqr}={hys}_{i}+p_{j}+c_{k}+m_{l}+{afc}_{m}+{\mathrm{DIM}}_{n}+a_{p}+{pe}_{q}+e_{ijklmnpqr} \end{array}$$ (2)

where $y_{ijklmnpqr}$ are the phenotypic records for DML and MLF, ${\mathrm{DIM}}_{n}$ is the fixed effect of lactation stage at the beginning of the ML (four levels, including 1–44 days, 45–99 days, 100–199 days, and 200–305 days); ${pe}_{q}$ is the random permanent environmental effect with *p* $e\;∼\;N\left(0,\;I\sigma_{pe}^{2}\right)$ . Other fixed and random effects are the same as in the single-trait model.

The genetic correlations between all pairs of resilience indicators were calculated based on bivariate animal models. The bivariate-trait animal model included the same effects as the single-trait model. The assumptions of additive genetic and the residual effects are:

$$\left[\begin{array}{c} a_{1}\\ a_{2} \end{array}\right]\;∼\;N\left[\left(\begin{array}{c} 0\\ 0 \end{array}\right),\;A⊗\left(\begin{array}{cc} \sigma_{a_{1}}^{2} & \sigma_{a_{1}a_{2}}\\ \sigma_{a_{1}a_{2}} & \sigma_{a_{2}}^{2} \end{array}\right)\right]$$

$$\left[\begin{array}{c} e_{1}\\ e_{2} \end{array}\right]\;∼\;N\left[\left(\begin{array}{c} 0\\ 0 \end{array}\right),\;I⊗\left(\begin{array}{cc} \sigma_{e_{1}}^{2} & \sigma_{e_{1}e_{2}}\\ \sigma_{e_{1}e_{2}} & \sigma_{e_{2}}^{2} \end{array}\right)\right]$$

where $a_{i}$ is the additive genetic effects for trait i , $\sigma_{a_{i}}^{2}$ is the additive genetic variance of trait i , $\sigma_{a_{i}a_{j}}$ is the additive genetic covariance between trait i and j , $e_{i}$ is the residual effect for trait i , $\sigma_{e_{i}}^{2}$ is the residual variance of trait i , $\sigma_{e_{i}e_{j}}$ is the residual covariance between trait i and j . The heritability, genetic correlations, and reliability of the estimated breeding value (EBV) for each trait were calculated as described in Su et al. (2007) and Luo et al. (2021).

#### 2.5.2 Genetic correlation with milk production, reproduction, longevity, and health traits

Genetic correlations between resilience indicators with economically important traits included milk production, reproduction, longevity, and health traits were calculated based on bivariate animal models. The milk production trait refers to MY305 calculated in this study. For the milk production trait and resilience indicators, the animal models used are the same as model [1]. For the five reproduction traits, the fixed effects included in the models were herd-year of measurement, parity and calving season, and the random effects of animal additive genetic, permanent environment, and residual effects, which are detailed in Guo et al. (2014) and Liu et al. (2017). For the three longevity traits, the fixed effects of age at first calving, herd-year of birth, and birth season and the random effect of additive genetic and residual effects were fitted and are detailed in Zhang et al. (2021). Furthermore, for the four health traits, herd-year of measurement, parity, and calving season were fitted as fixed effects in the model and animal additive genetic, permanent environment, and residual as random effects, as detailed in Wang et al. (2022). These analyses were implemented using the DMU software (Madsen et al., 2006).

### 2.6 Validation

To validate the resilience indicators evaluated in this study and determine whether selection on these indicators can improve the “true” resilience of offspring, thirty-four bulls with at least 40 daughters in first parity with divergent resilience indicators were retained. For each bull, the daughters were divided in prediction and validation datasets based on their birth date with allocation of 80% (older) and 20% (younger) of the animals in the prediction (*n* = 2,566) and validation (*n* = 641) datasets, respectively. The EBV of the resilience indicators for each cow in the validation dataset were estimated based on the phenotypes of the prediction dataset and pedigree information, and the model was the same as model 1. In total, the top and bottom 20% resilient animals were selected based on their EBV for each resilience indicator. The differences in EBV for production, reproduction, longevity, and health traits between the top and bottom resilience EBVs were statistically compared based on a Student *t* test.

## 3 Results

### 3.1 Lactation clustering and lactation curves

The descriptive statistics for the lactation clusters of the final dataset are presented in Supplemental Table S4. The final number of lactations in each cluster group was reduced from the number presented in Figure 1, but the order of numbers of lactations and the trend of daily milk yield within cluster groups did not change. The largest cluster [group (a)] included 3,877 lactations (43.39%), while the smallest cluster [group (f)] contained 16 lactations (0.18%). The differences of lactation curves among the six cluster groups mainly focused on parity, peak day, peak yield, and lactation persistency. The average parity for groups (a), (b), (c), (d), (e), and (f) was 2.67 ± 1.11, 2.12 ± 1.17, 2.03 ± 1.18, 1.37 ± 0.85, 1.36 ± 0.83, and 2.12 ± 1.18, respectively. The highest peak yield was in group (a) (48.27 ± 10.40 kg), with 10.80 kg difference from the lowest group [group (e), 37.47 ± 6.94 kg]. The peak day in group (a), (b), (c), and (f) was at the early lactation period (DIM 1–99), while the peak day in groups (d) and (e) was at the mid lactation (DIM 100–199). The latest peak day was observed for group (e) with 172.14 ± 57.40 days. For the three groups with the highest number of lactations, the groups (a) and (b) presented the highest average parity, normal peak day, and a clear downward phase after peak day, which is more representative of multiparous cows’ lactation curve. The group (d) presented a lower average parity and lower peak yield and slower decline in late lactation than groups (a) and (b), which represents the majority of primiparous cows. The groups (c), (e), and (f) exhibited an atypical pattern (5.3%) characterized by higher milk yield in early lactation, a delayed lactation peak, or a slower decline of milk yield in late lactation, while some reversal shaped curves and continuously increasing curve were also included in these groups.

The comparisons of four lactation curve models are presented in Supplemental Table S5. There were 5,137 lactations with the Ali-Schaeffer model as the optimal model in fitting ELC, accounting for 57.49%. While the Nelder model included the lowest number of lactations (731 lactations). After the iterative procedure and quality control, the average amount of data used to predict the ELC was 283.02 ± 14.76, and the average R^2^ of the ELC was 0.89 ± 0.06. The major difference between the four models was the percentage of the first parity. There were 66.94%, 82.17%, 31.15%, and 37.03% of lactations in which the first parity data were fitted with Wood, Nelder, Wilmink, and Ali-Schaeffer model, respectively.

In this study, cluster group and lactation curve model had a significant effect (*P* < 0.05) on milk loss traits and variability traits. The least squares mean estimates (LSM) of various levels on ML and Lnsd2 and multiple comparisons based on Bonferroni *t* corrected are presented in Supplemental Table S6. The LSM of ML and Lnsd2 in group (c) and (f) were significantly higher than that in other groups (*P* < 0.05), and the ML and Lnsd2 were lowest in group (a). For the lactation curve model, the ELC calculated by Ali-Schaeffer model had the highest ML and Lnsd2, whereas the lowest ones were calculated by Wilmink model.

### 3.2 Descriptive statistics and genetic parameters of resilience indicators

The distributions of MY305, milk loss traits, and variability traits are presented in Supplemental Figure S1. MY305, NML, and TDML were normally distributed and other milk loss traits (ML, MLP, DML, and MLF) showed a right skewed distribution. The variability traits had different distribution characteristics in the four periods evaluated. All four Lnsd variables were normally distributed and the four Ra variables showed a left skewed distribution. Ske1, Ske2, and Ske4 were left skewed while Ske3 was right skewed.

**FIGURE 4:**
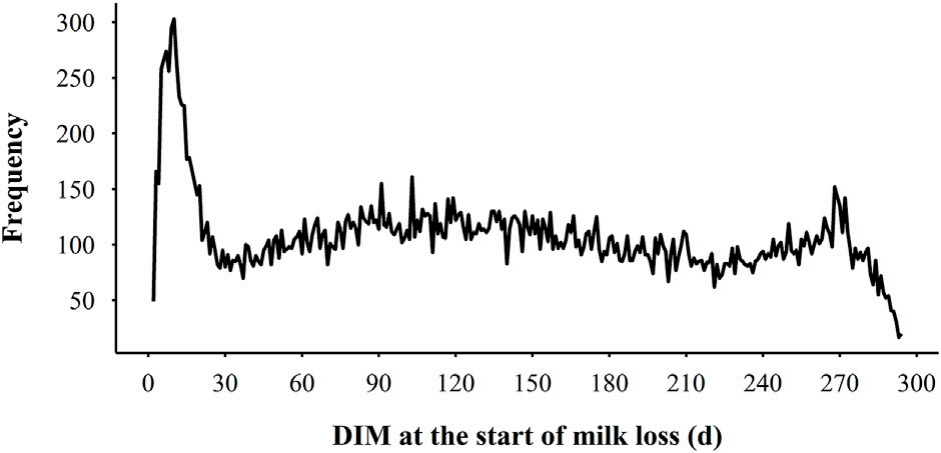
The distribution of days in milk (DIM) at the start of milk loss in each lactation.

The descriptive statistics for MY305, milk loss traits, and variability traits are presented in Table 1. MY305 ranged from 2,534.32 kg to 17,845.34 kg, with an average of 9,603.12 ± 2,354.72 kg. In general, cows experienced 3.73 ± 1.37 perturbations per lactation, ranging from 0 to 9. Cows in parity 1, 2, and 3 + experienced 3.70 ± 0.02, 3.76 ± 0.03, and 3.78 ± 0.03 perturbations per lactation, respectively. Only 32 lactations (0.36%) had no perturbations, while 3.54%, 14.08%, 26.98%, 27.53%, and 27.51% lactations had 1, 2, 3, 4, and 5 or more perturbations, respectively. The average TDML was 73.12 ± 26.42 days, with an average ML of 267.00 ± 185.04 kg (2.90% to average MY305). For the cows with the most severe milk loss, the TDML was 221 days, with ML of 2,170.31 kg (30.47% of average MY305). For each perturbation, the average DML was 19.80 ± 11.46 days and MLF was 67.48 ± 77.80 kg on average. The coefficient of variation for DML and MLF was 57.88% and 115.29%, respectively. The highest MLF was 1,989.74 kg, which lasted for 167 days. The distribution of DIM at the beginning of ML is presented in Figure 4. There were larger risks for ML from DIM 5–15, DIM 90–110, and DIM 270 and greater based on the prevalence of variability, while lower risks in mid-late lactation stage (DIM 120–250). The greatest risk of ML was in early lactation, with 12.73% ML events beginning within the first 20 days after calving. The average Lnsd1 was 1.11 ± 0.41, which meant the range of the 95% confidence interval for the deviation of actual milk yield from the expected values was ±5.94 kg. Among the Lnsd variables, the largest and lowest variation was observed for Lnsd4 and Lnsd1 with a coefficient of variation of 58.97% and 36.94%, respectively. Among the four Ra variables, the highest mean value was Ra2 (0.87) which was 0.04–0.10 higher than the other Ra, and its minimum value was 0.66 (0.2–0.3 higher than the other Ra variables). The coefficient of variation for Ra variables was small, with the highest being Ra4 (12.99%) and the lowest being Ra2 (5.74%). The average of four Ske variables were all less than 0. Ske3 had the highest average of −0.68 ± 0.76 and Ske1 had the lowest average of −1.82 ± 1.92. The variation of the four Ske variables was quite different, with the coefficient of variation ranging from 36.31% to 111.76%.

**TABLE 1:** Descriptive statistics of 305 days milk yield and resilience indicators in Chinese Holstein cattle.

Trait^1^	N	Mean	SD	Min	Max	Coefficient of variation
MY305, kg	8,935	9,603.12	2,354.72	2,534.32	17,845.34	24.52
NML, time	8,935	3.73	1.37	0	9	36.73
TDML, d	8,935	73.12	26.42	0	211	36.13
ML, kg	8,935	267.00	185.04	0.00	2,170.31	69.30
MLP, %	8,935	2.90	2.14	0.00	30.47	73.79
DML, d	31,606	19.80	11.46	10	167	57.88
MLF, kg	31,606	67.48	77.80	2.82	1,989.74	115.29
Lnsd1	8,935	1.11	0.41	−0.01	2.62	36.94
Lnsd2	8,935	0.97	0.38	−0.11	2.41	39.18
Lnsd3	8,935	0.89	0.44	−0.47	2.65	49.44
Lnsd4	8,935	0.78	0.46	−0.52	2.38	58.97
Ra1	8,935	0.83	0.08	0.36	0.98	9.64
Ra2	8,935	0.87	0.05	0.66	0.99	5.74
Ra3	8,935	0.83	0.07	0.46	0.99	8.43
Ra4	8,935	0.77	0.10	0.37	0.98	12.99
Ske1	8,935	−1.82	1.92	−10.65	6.81	105.49
Ske2	8,935	−0.96	0.79	−4.97	3.29	82.29
Ske3	8,935	−0.68	0.76	−3.24	4.18	111.76
Ske4	8,935	−1.57	0.57	−5.16	0.10	36.31

^1^ N, the number of records or indicators; MY305, 305 days milk yield; NML, number of milk loss events; TDML, total number of days for milk loss per lactation; ML, sum of the milk yield which dropped in all fluctuation phases in a lactation; MLP, the percentage of ML, to MY305; DML, length of each milk loss period in days; MLF, milk loss in each milk loss period; Lnsd, log-transformed standard deviation of milk deviations; Ra, lag-1, autocorrelation of milk deviations; Ske, skewness of milk deviations. These three variability traits were calculated based on records from the entire lactation (Lnsd1, Ra1, and Ske1, from DIM 1–305), lactation period when removing the first and last 10 days (Lnsd2, Ra2, and Ske2, from DIM 11–295), during the lactation peak period (Lnsd3, Ra3, and Ske3, from DIM 60–90), and the period consisting of each DIM, when the actual milk yield was below the expected lactation curve (ELC) fitted value (Lnsd4, Ra4, and Ske4), respectively.

Estimates of variance components and heritability for MY305, milk loss traits, and variability traits are presented in Table 2. The heritability for milk loss traits ranged from 0.004 ± 0.003 (DML) to 0.061 ± 0.016 (ML), all of which had low heritability estimates. All four Lnsd variables had moderate heritability estimates (from 0.135 to 0.250). Lnsd2 had the highest heritability at 0.250 ± 0.021, followed by Lnsd4 at 0.184 ± 0.021. Similar heritability estimates were observed for Lnsd1 and Lnsd3. The heritabilities for Ra and Ske were all below 0.10, ranging from 0.001 ± 0.005 (Ske2) to 0.075 ± 0.016 (Ske1). Ra1 (0.058 ± 0.015) and Ske1 (0.075 ± 0.016) had the highest heritability estimates among Ra and Ske variables.

**TABLE 2:** Estimates of additive genetic variance ( ${\hat{\sigma }}_{a}^{2}$ ), permanent environment variance ( ${\hat{\sigma }}_{pe}^{2}$ ), residual variance ( ${\hat{\sigma }}_{e}^{2}$ ), and heritability ( $\hat{h^{2}}$ ) for 305 days milk yield, milk loss traits, and variability traits.

Trait^1^	N	${\hat{\sigma }}_{a}^{2}\;\left({\hat{\sigma }}_{pe}^{2}\right)$	${\hat{\sigma }}_{e}^{2}$	$\hat{h^{2}}$
MY305, kg	8,935	861,242.143	2,704,320.109	0.242 ± 0.036
NML, time	8,935	0.043	1.740	0.024 ± 0.010
TDML, d	8,935	9.155	647.551	0.014 ± 0.008
ML, kg	8,935	1,758.075	26,958.841	0.061 ± 0.016
MLP, %	8,935	0.182E-04	0.397E-03	0.044 ± 0.013
DML^2^, d	31,606	0.546 (0.698)	125.352	0.004 ± 0.003
MLF, kg	31,606	29.506 (167.004)	5,430.781	0.005 ± 0.003
Lnsd1	8,935	0.016	0.100	0.137 ± 0.020
Lnsd2	8,935	0.027	0.080	0.250 ± 0.020
Lnsd3	8,935	0.020	0.131	0.135 ± 0.020
Lnsd4	8,935	0.031	0.137	0.184 ± 0.020
Ra1	8,935	0.304E-03	0.495E-02	0.058 ± 0.015
Ra2	8,935	0.539E-04	0.187E-02	0.028 ± 0.011
Ra3	8,935	0.578E-04	0.407E-02	0.014 ± 0.008
Ra4	8,935	0.809E-04	0.975E-02	0.008 ± 0.007
Ske1	8,935	0.221	2.732	0.075 ± 0.016
Ske2	8,935	0.496E-03	0.553	0.001 ± 0.005
Ske3	8,935	0.019	0.495	0.037 ± 0.012
Ske4	8,935	0.004	0.304	0.013 ± 0.008

^1^ N, the number of records or indicators; MY305, 305 days milk yield; NML, number of milk loss events; TDML, total number of days for milk loss per lactation; ML, sum of the milk yield which dropped in all fluctuation phases in a lactation; MLP, the percentage of ML to MY305; DML, length of each milk loss period in days; MLF, milk loss in each milk loss period; Lnsd, log-transformed standard deviation of milk deviations; Ra, lag-1 autocorrelation of milk deviations; Ske, skewness of milk deviations. These three variability traits were calculated based on records from the entire lactation (Lnsd1, Ra1, and Ske1, from DIM 1–305), lactation period when removing the first and last 10 days (Lnsd2, Ra2, and Ske2, from DIM 11–295), during the lactation peak period (Lnsd3, Ra3, and Ske3, from DIM 60–90), and the period consisting of each DIM when the actual milk yield was below the expected lactation curve (ELC) fitted value (Lnsd4, Ra4, and Ske4), respectively.

The genetic correlations among the variability traits are presented in Table 3. The genetic correlations within each trait were high among the four periods. For instance, the genetic correlations among the four Lnsd variables ranged from 0.93 ± 0.02 to 0.99 ± 0.00, and among the four Ra variables ranged from 0.69 ± 0.17 to 0.99 ± 0.12. Within each lactation period, the genetic correlations across the variability traits were not consistent. The genetic correlations between Lnsd and Ra were positive across the different periods, with a minimum of 0.28 ± 0.14 (Lnsd1 and Ra1) and a maximum of 0.77 ± 0.06 (Lnsd2 and Ra2). The genetic correlations between Lnsd and Ske as well as Ra and Ske varied considerably across lactation periods. For instance, positive genetic correlations were observed in the first (between Lnsd1 and Ske1; and, Ra1 and Ske1) and fourth periods (between Lnsd4 and Ske4; and, Ra4 and Ske4) and negative correlations in the third period (between Lnsd3 and Ske3; and, Ra3 and Ske3).

**TABLE 3:** Genetic (r_G_) and phenotypic (r_P_) correlations among variability traits^1^.

	Lnsd1	Lnsd2	Lnsd3	Lnsd4	Ra1	Ra2	Ra3	Ra4	Ske1	Ske2	Ske3	Ske4
Lnsd1^2^		0.93 (0.02)	0.96 (0.02)	0.93 (0.02)	0.28 (0.14)	0.43 (0.15)	0.62 (0.15)	0.38 (0.25)	0.09 (0.13)	—	−0.61 (0.12)	0.45 (0.24)
Lnsd2	0.79 (0.00)		0.99 (0.01)	0.99 (0.00)	0.70 (0.07)	0.77 (0.06)	0.76 (0.10)	0.70 (0.14)	0.51 (0.10)	—	−0.68 (0.09)	0.74 (0.17)
Lnsd3	0.57 (0.01)	0.74 (0.01)		0.99 (0.01)	0.67 (0.10)	0.15 (0.28)	0.50 (0.16)	0.57 (0.19)	0.35 (0.13)	—	−0.75 (0.10)	0.85 (0.17)
Lnsd4	0.76 (0.00)	0.93 (0.00)	0.67 (0.01)		0.63 (0.09)	0.70 (0.08)	0.73 (0.12)	0.54 (0.16)	0.56 (0.11)	—	−0.67 (0.10)	0.74 (0.20)
Ra1	0.15 (0.01)	0.49 (0.01)	0.31 (0.01)	0.47 (0.01)		0.78 (0.10)	0.69 (0.17)	0.77 (0.19)	0.88 (0.06)	—	−0.09 (0.21)	0.81 (0.18)
Ra2	0.45 (0.01)	0.66 (0.01)	0.43 (0.01)	0.62 (0.01)	0.57 (0.01)		0.80 (0.16)	0.99 (0.12)	0.61 (0.15)	—	0.15 (0.28)	0.54 (0.28)
Ra3	0.24 (0.01)	0.35 (0.01)	0.60 (0.01)	0.32 (0.01)	0.29 (0.01)	0.50 (0.01)		0.74 (0.32)	0.32 (0.24)	—	−0.37 (0.29)	0.50 (0.35)
Ra4	0.46 (0.01)	0.60 (0.01)	0.37 (0.01)	0.70 (0.01)	0.48 (0.01)	0.82 (0.00)	0.40 (0.01)			—	—	0.53 (0.46)
Ske1	0.46 (0.01)	0.09 (0.01)	0.07 (0.01)	0.03 (0.01)	0.48 (0.01)	0.09 (0.01)	0.04 (0.01)	0.05 (0.01)		—	−0.17 (0.19)	0.61 (0.19)
Ske2	--^3^	—	—	—	—	—	—	—	—		—	—
Ske3	0.15 (0.01)	0.17 (0.01)	0.21 (0.01)	0.30 (0.01)	0.05 (0.01)	0.12 (0.01)	0.18 (0.01)	—	0.13 (0.01)	—		−0.78 (0.31)
Ske4	0.07 (0.01)	0.05 (0.01)	0.11 (0.01)	0.16 (0.01)	0.05 (0.01)	0.09 (0.01)	0.07 (0.01)	0.23 (0.01)	0.29 (0.00)	—	0.14 (0.01)	

^1^ The genetic correlations are presented above the diagonal while the phenotypic correlations are below the diagonal.

^2^ Lnsd, log-transformed standard deviation of milk deviations; Ra, lag-1, autocorrelation of milk deviations; Ske, skewness of milk deviations. These three variability traits were calculated based on records from the entire lactation (Lnsd1, Ra1, and Ske1, from DIM 1–305), lactation period when removing the first and last 10 days (Lnsd2, Ra2, and Ske2, from DIM 11–295), during the lactation peak period (Lnsd3, Ra3, and Ske3, from DIM 60–90), and the period consisting of each DIM, when the actual milk yield was below the expected lactation curve (ELC) fitted value (Lnsd4, Ra4, and Ske4), respectively.

^3^ -- means that the analyses did not converge.

The genetic correlations among the milk loss traits and between milk loss traits and variability traits are presented in Table 4. The three traits with the highest heritability among the three variability traits (Lnsd2, Ra1, and Ske1) are presented. Positive genetic correlations were observed between different milk loss traits, ranging from 0.21 ± 0.21 (ML and NML) to 0.78 ± 0.13 (TDML and MLP). Lnsd2 and Ra1 had positive genetic correlations with milk loss traits, ranging from 0.09 ± 0.32 (Ra1 and TDML) to 0.96 ± 0.01 (Lnsd2 and ML), with the exception of Ske1 which had mostly negative genetic correlations. However, only Lnsd2 had statistically significant genetic correlations with all four milk loss traits at the 5% level. There were moderate to high genetic correlations between Lnsd2 and all milk loss traits, ranging from 0.45 ± 0.14 (NML) to 0.96 ± 0.01 (ML).

**TABLE 4:** Genetic and phenotypic correlations among milk loss traits and genetic correlations between milk loss traits and variability traits.

s	N^2^	ML	NML	TDML	MLP	Lnsd2	Ra1	Ske1
ML, kg	8,935		0.21 (0.21)	0.29 (0.23)	0.48 (0.13)	0.96 (0.01)	0.62 (0.13)	0.51 (0.16)
NML, time	8,935	0.39 (0.01)		0.69 (0.20)	0.58 (0.18)	0.45 (0.14)	0.23 (0.24)	−0.27 (0.21)
TDML, d	8,935	0.66 (0.01)	0.67 (0.01)		0.78 (0.13)	0.58 (0.14)	0.09 (0.32)	−0.41 (0.30)
MLP, %	8,935	0.89 (0.00)	0.39 (0.01)	0.67 (0.01)		0.54 (0.08)	0.16 (0.20)	−0.02 (0.18)

^1^The genetic correlations among milk loss traits are presented above the diagonal while the phenotypic correlations are below the diagonal in the first four columns, while the genetic correlations between milk loss traits and variability traits are presented in the last three columns; ML, sum of the milk yield which dropped in all fluctuation phases in a lactation; NML, number of milk loss events; TDML, total number of days for milk loss per lactation; MLP, the percentage of ML to MY305; Lnsd2, log-transformed standard deviation of milk deviations based on the lactation when removing first and last 10 DIM; Ra1, lag-1 autocorrelation of milk deviations based on the entire lactation; Ske1, skewness of milk deviations based on the entire lactation. Lnsd1, Ra2 and Ske2 are the traits with the highest heritability among the three variability traits, respectively.

^2^N: number of records that were used to calculate the genetic correlations.

### 3.3 Genetic correlation with milk production, reproduction, longevity, and health traits

The genetic correlations of resilience indicators with production, reproduction, longevity, and health traits are presented in Table 5. The genetic correlations of DML and MLF with routinely evaluated traits are not presented because the analyses did not converge.

**TABLE 5:** Genetic correlations between milk loss traits, variability traits and production, reproduction, longevity, and health traits.

s	N^2^	ML	NML	TDML	MLP	Lnsd2	Ra1	Ske1
MY305, kg	8,935	0.60 (0.08)	−0.46 (0.14)	−0.75 (0.15)	−0.65 (0.11)	0.80 (0.04)	0.56 (0.10)	0.53 (0.09)
AFC, d	4,222	0.25 (0.05)	0.04 (0.10)	0.03 (0.07)	0.64 (0.29)	0.32 (0.03)	0.31 (0.07)	−0.17 (0.06)
AFS, d	4,222	−0.22 (0.39)	0.28 (0.41)	0.87 (0.42)	−0.05 (0.37)	0.05 (0.03)	0.19 (0.07)	0.37 (0.07)
IFL_H, d	3,871	−0.86 (0.66)	0.34 (0.64)	−0.49 (0.83)	−0.91 (0.42)	0.12 (0.03)	−0.11 (0.11)	−0.19 (0.06)
IFL_C, d	4,476	−0.16 (0.36)	0.25 (0.42)	−0.88 (0.96)	−0.32 (0.35)	0.59 (0.20)	−0.02 (0.44)	0.12 (0.32)
ICF, d	8,860	0.41 (0.18)	0.38 (0.21)	0.29 (0.27)	0.48 (0.18)	0.19 (0.11)	0.14 (0.18)	0.49 (0.17)
Lon1, d	2,610	0.16 (0.17)	−0.16 (0.17)	0.02 (0.11)	−0.97 (0.14)	0.24 (0.09)	0.18 (0.13)	0.19 (0.15)
Lon2, d	1,350	0.01 (0.15)	−0.35 (0.16)	−0.19 (0.13)	−0.96 (0.32)	0.02 (0.10)	0.08 (0.15)	0.28 (0.13)
PL, d	883	0.05 (0.25)	−0.08 (0.10)	−0.16 (0.23)	−0.96 (0.74)	0.49 (0.23)	0.19 (0.15)	0.16 (0.17)
UDDE	7,347	0.58 (0.18)	0.18 (0.30)	0.21 (0.41)	0.19 (0.24)	0.87 (0.07)	0.84 (0.14)	0.62 (0.18)
REPR	7,347	0.66 (0.21)	0.49 (0.24)	0.69 (0.31)	0.70 (0.19)	0.36 (0.22)	0.30 (0.25)	0.52 (0.19)
METB	5,921	−0.49 (0.27)	−0.55 (0.29)	--^3^	0.01 (0.33)	−0.47 (0.19)	−0.64 (0.25)	−0.95 (0.23)
DIGS	5,921	−0.87 (1.77)	−0.88 (1.94)	—	−0.87 (1.96)	0.05 (0.89)	−0.55 (1.29)	−0.33 (1.34)

^1^MY305, 305 days milk yield; ML, sum of the milk yield which dropped in all fluctuation phases in a lactation; NML, number of milk loss events; TDML, total number of days for milk loss per lactation; MLP, the percentage of ML to MY305; AFC, age at first calving in heifers; AFS, age at first insemination in heifers; IFL_H, interval from first to last insemination in heifers; IFL_C, interval from first to last insemination in cows; ICF, interval from calving to first insemination; Lon1, the days from the first calving to the end of the first lactation or culling; Lon2, the days from the first calving to the end of the second lactation or culling; PL, productive life referring the days from the first calving to culling or death; UDDE, udder health; REPR, reproductive disorders; METB, metabolic disorders; DIGS: digestive disorders; Lnsd2, log-transformed standard deviation of milk deviations based on the lactation when removing first and last 10 DIM; Ra1, lag-1 autocorrelation of milk deviations based on the entire lactation; Ske1, skewness of milk deviations based on the entire lactation. Lnsd1, Ra2, and Ske2 are the traits with the highest heritability among the three variability traits, respectively.

^2^N: number of records that were used to calculate the genetic correlations.

^3^-- means that the analyses did not converge.

The estimated genetic correlations between milk loss traits (NML, TDML, and MLP) and MY305 were negative and ranged from −0.46 ± 0.14 (NML) to −0.75 ± 0.15 (TDML), except for a positive genetic correlation between ML and MY305 (0.60 ± 0.08). The genetic correlation between variability traits and MY305 were positive and ranged from 0.53 ± 0.09 (Ske1) to 0.80 ± 0.04 (Lnsd2).

The estimated genetic correlations between milk loss traits, variability traits and reproduction, longevity, and health traits were mostly moderate to high while most of them were not significantly different from zero because of the high standard errors. There were favorable and unfavorable genetic correlations for ML with AFC (0.25 ± 0.05), AFS (−0.22 ± 0.39), IFL_H (−0.86 ± 0.66), IFL_C (−0.16 ± 0.36), and ICF (0.41 ± 0.18), while the genetic correlations between NML and reproduction traits were all positive. For variability traits, the genetic correlations between Lnsd2 and reproduction traits were positive and ranged from 0.05 ± 0.03 (AFS) to 0.59 ± 0.20 (IFL_C), and were all statistically significant at the 5% level. However, for the other two variability traits, the genetic correlations ranged from −0.19 ± 0.06 (Ske1 and IFL_H) to 0.49 ± 0.17 (Ske1 and ICF). There were negative genetic correlations between NML and Lon2, TDML and Lon2, MLP and all longevity traits, ranging from -0.97 ± 0.03 (MLP and Lon1) to −0.19 ± 0.03 (TDML and Lon2), whereas the other estimates between milk loss traits and longevity traits were not significantly different from zero. The genetic correlations between variability traits and longevity traits were positive and ranged from 0.02 ± 0.10 (Lnsd2 and Lon2) to 0.49 ± 0.23 (Lnsd2 and PL). Positive genetic correlations, ranging from 0.18 ± 0.30 (NML and UDDE) to 0.70 ± 0.19 (MLP and REPR) were obtained between milk loss traits and UDDE and REPR. The genetic correlations between milk loss traits and METB and DIGS were mostly unfavorable. Similar correlations were obtained in the genetic correlations between variability traits and health traits. Among all health traits, UDDE had the highest genetic correlation with Lnsd2 (0.87 ± 0.07). Among all genetic correlations with economically important traits, the standard errors were on average higher for the milk loss traits than for the variability traits and Lnsd2 had the lowest standard errors on average. For instance, the standard errors for estimates of genetic correlations between milk loss traits and reproduction traits ranged from 0.05 to 0.96, while the standard errors ranged from 0.03 to 0.44 for the variability traits.

### 3.4 Validation

The comparisons of the milk loss, production, reproduction, longevity, and health traits of the top and bottom 20% EBVs in the validation dataset for Lnsd2 are presented in Table 6. The results for Lnsd1, Lnsd3, and Lnsd4 are presented in Supplemental Tables S7–S9. The top 20% of Lnsd EBVs represent the 20% most resilient cows. The top 20% group was significantly better in MLP and 0.72% lower on average than the bottom 20% group. AFC, IFL_H, and Lon1 were significantly better in the top 20% group than in the bottom 20% group among the ten production, reproduction, longevity, and health traits. For AFC and IFL_H, the top 20% group was 17.83 and 17.08 days less than the bottom 20% group, respectively, and Lon1 was 14.98 days longer. However, for the other traits, although the differences between the two groups were not statistically significant, there was still a trend by most traits towards less milk loss, better productive performance, and lower disease incidence in the top 20% group (more resilient animals). For instance, ML was 22.92 kg lower, MY305 was 284.48 kg higher and UDDE was 2% lower in the top 20% group. Nevertheless, AFS, ICF, Lon2, and REPR showed a more favourable trend in the bottom 20% group.

**TABLE 6:** Comparison of top 20% and bottom 20% estimated breeding values (EBVs) in the validation dataset of Lnsd2.

Trait^1^	N	Top 20%	Bottom 20%	*P*-Value
EBV	128	−0.02 ± 0.01	0.04 ± 0.01	<0.01**
Lnsd2	128	0.84 ± 0.31	0.87 ± 0.34	0.26
MY305, kg	128	8,814.02 ± 1,876.79	8,529.54 ± 2,044.03	0.12
ML, kg	128	195.80 ± 126.37	218.72 ± 151.70	0.09
NML, time	128	3.53 ± 1.35	3.70 ± 1.42	0.16
TDML, d	128	68.60 ± 27.08	69.72 ± 27.58	0.74
MLP, %	128	2.27 ± 1.55	2.79 ± 2.32	0.02*
AFC, d	128	698.08 ± 35.77	715.91 ± 86.65	0.03*
AFS, d	128	415.23 ± 11.51	400.60 ± 22.71	<0.01**
IFL_H, d	119	14.92 ± 35.05	32.00 ± 54.43	<0.01**
ICF, d	125	66.48 ± 7.18	64.60 ± 6.60	0.03*
Lon1, d	65	380.00 ± 60.21	365.02 ± 10.35	0.03*
Lon2, d	22	664.91 ± 114.05	668.77 ± 135.81	0.54
UDDE	105	0.28 ± 0.46	0.30 ± 0.46	0.43
REPR	105	0.19 ± 0.39	0.17 ± 0.38	0.68
METB	119	0.03 ± 0.18	0.04 ± 0.20	0.38
DIGS	112	0.02 ± 0.16	0.02 ± 0.14	0.65

^1^ EBV, estimated breeding value; Lnsd2, log-transformed standard deviation of milk deviations based on the lactation when removing first and last 10 DIM; MY305, 305 days milk yield; ML, sum of the milk yield which dropped in all fluctuation phases in a lactation; NML, number of milk loss events; TDML, total number of days for milk loss per lactation; MLP, the percentage of ML to MY305; AFC, age at first calving in heifers; AFS, age at first insemination in heifers; IFL_H, interval from first to last insemination in heifers; ICF, interval from calving to first insemination; Lon1, the days from the first calving to the end of the first lactation or culling; Lon2, the days from the first calving to the end of the second lactation or culling; UDDE, udder health; REPR, reproductive disorders; METB, metabolic disorders; DIGS, digestive disorders.

## 4 Discussion

### 4.1 Analyses of longitudinal data

Traits with repeated records over time for the same individual are known as longitudinal traits (Ning et al., 2018; Oliveira et al., 2019), which can be expressed as a series of independent continuous functions (Pletcher and Geyer, 1999; Oliveira et al., 2019). When selecting a longitudinal trait to analyze resilience in cattle, there are several points to be considered. Firstly, the trait should be susceptible to monitorable fluctuations by environmental disturbances. Secondly, the time interval between record points should be less than the duration of the fluctuation (Mehrabbeik et al., 2021), otherwise the short-term fluctuations will not be captured. In the process of recording daily milk yield by automatic monitoring equipment, missing data would inevitably occur due to errors in identifying cows or recording. For lactations missing more than 10 consecutive days, it was assumed that the true fluctuations in that phase could not be known. Afterwards, the quality of raw records is an important factor. The two steps in the quality control which removed the most records were the number of records within a lactation and lactation curve, which caused removal of 19,836 and 9,780 lactations, respectively (the total number of lactations removed was 40,183). In our study, 42.2% lactations did not meet the threshold for the number of records within a lactation. Culling, damage to monitoring equipment, diseases, and a variety of other unknown reasons can result in missing records. In particular, when cows are not milked due to disease, data imputation in milk loss period cannot accurately reflect the disturbance. Matching the two types of data, milk yield and environment disturbance, could be beneficial when possible. To ensure the lactation curve, extreme values and R^2^ were controlled. This step is important because the empirical lactation model tends to be a quantitative representation of the phenomenon and therefore would be more susceptible to extremes (Macciotta et al., 2011). In addition to the observed phenotypic outliers, some daily milk yield records derived from the imputation analyses were also out of the expected range. The DIM 1–4 days and DIM 305 of data used for the data imputation were based on normalized values for different cluster groups. When the standard deviation of non-missing milk yield is too high, the values converted back to the original scale would likely be negative or too high. As a result, the proportion of extremes in our study was increased. The low R^2^ for some lactation curves may be due to the atypical shape, as discussed in Section 4.2. Finally, the methodology for analyzing longitudinal data should be precisely tailored to the characteristics of the data. In our study, a weighted moving average filter was established to effectively eliminate the effects of random fluctuations in the raw data (Poppe et al., 2020). This study serves as an example of the analysis of longitudinal data and provides a reference for the future processing of continuous datasets.

### 4.2 Lactation curve and perturbations

Lactation curve is a mathematical model used to describe the trend of daily milk yield in a lactation (Kong et al., 2018; Oliveira et al., 2019). There are individual differences in the shape of this variation in daily milk yield, and even atypical lactation curves, where the shape is completely opposite to the standard curve or shows a linear shape with no peak yield (Lee et al., 2020). These curves account for about 10–20% in a population (Macciotta et al., 2005; Lee et al., 2020). In previous studies, atypical curves have often been ignored or their influence on the overall dataset has been diluted by using average values. Approximately 5.3% of lactations in our population [groups (c), (e), and (f)] exhibited atypical patterns and a greater tendency for atypical curves in low parity cows. Lee et al. (2020) clustered lactation curves using the K-medoids and the proportion of atypical curves was 18%, with average parity of 1.25, which is similar to our results. The reason for identifying fewer atypical curves in our study may be the differences in the type of raw data. Peak yield can easily be missed by using only DHI records to estimate lactation curve (Rekik and Gara, 2004; Macciotta et al., 2005), making the curves unrepresentative of trends of the true daily milk yield. Meanwhile, when there is an abnormal record (too high or too low) in the DHI records, it can have a large influence on the lactation curve. The high milk loss and high Lnsd2 of group (c), (e), and (f) indicated that the occurrence of atypical lactation curves is unfavorable for milk production and resilience breeding. The significant effects of the four lactation curve models on resilience indicators also indicate individual differences in the lactation trend. The identification and application of atypical curves should also be considered in future studies.

In our study, ELC and milk loss were estimated through an iterative procedure. The inclusion of milk loss in the lactation curve is a reasonable modification of the model based on production reality. The assumption is that there is a theoretical production potential for cows that corresponds to their genetic potential, which may not be fully expressed due to various environmental disturbances (Ben et al., 2021). The high variability in ML suggests that fluctuations in daily milk yield may help to identify environmental disturbances and reflect their ability to adapt and resilience to disturbances (Dunne et al., 2018). The maximum TDML was 221 days, which means that 221 days of a lactation had not reached lactation potential, and the maximum DML was 167 days, indicating that the longest period of milk loss in the population was 167 days. This is uncommon and may be related with the low level of milk yield that do not match the trend of milk yield before milk loss occurred. This could also be an issue with the ELC fitted. Although we used four lactation curve models expecting to restore the lactation potential as much as possible, the models still do not fit the data perfectly. Therefore, milk loss can be further addressed by setting thresholds or changing to a more optimal model in future studies. Adriaens et al. (2021b) detected 3.8 perturbations within a lactation, with milk losses ranging from 0 to 29%, using a threshold of five consecutive days of milk losses. Ben et al. (2021) considered each negative deviation as a perturbation and obtained milk losses ranging from 2 to 19%. Milk loss does not occur with the same frequency at all lactation stages. As we set a higher threshold, disturbances of longer duration such as clinical health events (LeBlanc, 2020; Adriaens et al., 2021b) and reproductive events (Strucken et al., 2015) were likely the main reasons for the high probability of milk loss in early and late lactation. The threshold could affect the number of perturbations identified, with more milk loss periods detected when thresholds are reduced. However, it is less directional and may detect decreases in milk yield which last for a short number of days without any cause, which is potentially not what we expect. Therefore, additional studies on milk loss thresholds need to be performed, such as milk yield per shift and specific environmental disturbances.

### 4.3 Resilience in Holstein cattle

In the case of livestock, resilience is defined as “the capacity of the livestock to maintain their level of production under environmental disturbances or to recover rapidly to the state existing before exposure to a disturbance” (Colditz and Hine, 2016; Berghof et al., 2018). Several concepts related to resilience have been discussed in many studies: robustness (De La Torre et al., 2015), tolerance (Bishop, 2012), environmental sensitivity (Ehsaninia et al., 2019, 2020), and plasticity (Debat and David, 2001). Despite the wealth of research in humans (Feder et al., 2019), studies on resilience in livestock are still incipient and there is no clear distinction between these definitions in terms of similarities and differences and their research strategies. It is important to note that we focus on “general” resilience which is a comprehensive breeding goal and not only “specific” resilience (e.g., disease resilience, climatic resilience). When stressors exceed the threshold of the “general” resilience, the homeostasis of the livestock system is disrupted (van Dixhoorn et al., 2018) and performance will be forced to shift from one equilibrium to another (Nazarimehr et al., 2020). In this study, there was a decline in daily milk yield until it reduced to a minimum. Close to the minimum point, the rate of decline in daily milk yield will become slower, a phenomenon known as critical slowing down (Ren and Watts, 2015; Nazarimehr et al., 2020; Mehrabbeik et al., 2021). As a consequence of this phenomenon, the deviation between actual and expected milk yield and its variation will increase, and the autocorrelation between subsequent states will become increasingly tight (Ren and Watts, 2015; Scheffer et al., 2018; van Dixhoorn et al., 2018). Therefore, the standard deviation (Lnsd), autocorrelation (Ra), and skewness (Ske) of the deviations have been proposed as resilience indicators in a complex dynamic system. Lnsd reflects the amplitude of fluctuation in daily milk yield within a lactation. We applied Ln transformation to the standard deviation to make the indicator normally distributed. The smaller the Lnsd, the lower the fluctuation in milk yield, indicating that the cow is less susceptible to environmental disturbance and therefore, more resilient. Ra reflects the length and rate of variation of the milk loss within a lactation. Resilient cows have greater independence between milk yield from successive DIM and therefore, smaller Ra. Ske reflects the balance of positive and negative deviations, with a high Ske indicating low milk loss. In addition, genetic selection for resilience by milk loss traits to reduce milk loss of cows in the general environment seems to be a potential direction which we explored in this study. Several studies have proposed other potential indicators, such as the rate of recovery (Adriaens et al., 2021b), the slope of the reaction norm (Kause and Odegård, 2012), and the cross-correlation between different longitudinal traits (Scheffer et al., 2018).

The best resilience indicators should have high heritability and be genetically correlated with better production, reproduction, longevity, and health traits (Poppe et al., 2020). When high heritability resilience indicator is applied for genetic selection, the accuracy of estimated breeding value as well as genomic selection can be ensured, while genetic antagonism resulting from selection for resilience causing a decrease in milk yield can be avoided as much as possible. Therefore, the selection of appropriate resilience indicators in this study was based on heritability and genetic correlation with economically important traits. Genetic correlations among the four milk loss traits were all positive. Higher ML, more NML, longer TDML, and higher MLP tended to coincide, which showed the overall consistency of milk loss traits. These traits represent the fluctuation of daily milk yield from different perspectives when cows face environmental perturbations. However, the highest heritability estimate was only 0.06 (ML) among milk loss traits which is low. In this context, using milk loss traits for breeding is less efficient. Milk loss traits are favorably genetically associated with several production, reproduction, longevity, and health traits, and in particular the high positive genetic correlations between ML and UDDE and REPR indicate that these health traits might be major causes of fluctuations (decreases) in daily milk yield. There was no clear pattern of genetic correlation between milk loss traits and economically important traits, and the accuracy of the correlation estimates was poor, with standard errors higher than estimates in some cases which were on average higher than the standard errors for the variability trait. This is unfavorable for the genetic selection for resilience through milk loss traits. The high standard errors might be due to the small data size used to estimate genetic correlations, and the complex distribution of phenotypes in different traits, especially for health traits. The low incidence would result in imbalanced binary phenotypes which might also have obstructed the accurate estimation of genetic correlations. A larger data size is required to further determine the relationship between milk loss traits and economically important traits. The genetic correlations between milk loss traits and MY305 were not consistent. The negative genetic correlations between NML, TDML, and MLP and MY305 indicated that fewer milk losses, shorter milk loss duration, and lower milk loss ratios all contributed to higher milk production, as expected. In contrast, the positive genetic correlation between ML and MY305 might be due to scale effects. When high yielding cows experience the same extent of environmental disturbances as low yielding cows, and milk production drops by the same percentage, the absolute value of milk loss is greater in high yielding cows and therefore, ML tends to be greater in high yielding cows. Nevertheless, the absolute amount of ML is important, and it is more necessary to minimize milk loss on high yielding cows to improving herd profitability, rather than focusing on the relative percentage of ML. Therefore, as new traits directly related to milk yield, milk loss traits should be further evaluated, especially using complete datasets with less missing records.

In this study, four periods of variability traits showed different genetic characteristics. The heritability of Lnsd was higher than the heritability of ML, whereas the heritabilities of Ra and Ske were much lower than that of ML. Poppe et al. (2020) obtained heritability estimates of 0.08–0.10 for Ra (higher than this study) and 0.01–0.02 for Ske (lower than this study). The lower heritability for Ra in this study may be due to the establishment of the 2-sided weighted moving average filter which might have removed part of the variability from the deviations. This approach resulted in more similarity between the deviations of successive DIMs, but the natural correlation was broken. The higher heritability for Ske was due to quality control. Ske was too sensitive to extreme milk yield (Poppe et al., 2020). In our study, Ske was more stable and representative due to the strict quality control and fitting procedures. Although these three variability traits referred to different aspect of resilience by definition, the moderate to high genetic correlations between the three highest heritability variability traits (Lnsd2, Ra1, and Ske1) showed that they contain overlapping information on resilience. Lnsd2, which characterizes the amplitude of fluctuation, is also representative of the information about the length of milk loss periods (as presented by Ra) and the negative deviations of milk loss (as presented by Ske). Ra and Ske also provide research value and characterize specific information about resilience. Berghof et al. (2018) pointed that a higher Ra was expected to indicate a slower recovery. However, the results of our study do not provide information on this aspect and individual milk loss require further validations. The reasons for differences in heritability of Lnsd are not the same for various periods. The lactation curves were poorly fitted during the early and late lactation because the raw data were more severely missing in these two periods, particularly when DIM was 1–10 and 296–305. Meanwhile, due to the high sensitivity of the Ali-Schaeffer model to data distribution (Melzer et al., 2017), the curves may take on an abnormally shape when there are episodic extreme values in the records during the early and late lactation. Therefore, calculating variability traits from entire lactation gave poor results. In addition, it may also be inappropriate to use only peak lactation period data due to the lower frequency of milk loss in mid-lactation. For the fourth period, as DIM with positive deviations were not included in the calculation, Lnsd would be based on the negative deviations. However, the number of negative deviations within each lactation is not the same, which results in the calculation of Lnsd not being based on the same scale of data volume and comparability becomes poor. For instance, when only 1 day is in negative deviation, the standard deviation is zero regardless of the amount of milk loss. Therefore, Lnsd2, which has the highest heritability, is the most suitable as a single resilience indicator.

Lower Lnsd2 was correlated with lower milk loss, better reproductive performance, and lower disease incidence at the genetic level with the smaller standard errors than other resilience indicators. The results of validation for Lnsd2 also supported this trend, although the results of the *t*-test were not all statistically significant. These results supported Lnsd2 as a potential resilience indicator. The moderate to high genetic correlations of Lnsd2 with milk loss traits indicate that Lnsd2 can characterize most aspects of milk loss with high genetic correlation of 0.96. Genetic selection for resilience by Lnsd2 is almost completely representative of selection directly by ML and is more efficient. Among the reproduction traits, AFS was less genetically correlated because the age at first insemination tends to be consistent in the herd and phenotypic variation is smaller than other reproduction traits (as presented in Supplemental Table S1). In contrast, all other reproduction traits associated with insemination showed significant genetic correlations with Lnsd2, indicating a strong effect of insemination success on daily milk yield. The genetic correlation between Lnsd2 and UDDE was 0.87. Thus, it is possible that a large part of fluctuations is caused by mastitis. Mastitis-associated milk losses have a large impact on milk yield and herd sustainability. Adriaens et al. (2021a) indicated that milk losses ranged from 38.4 to 215.6 kg within -5–30 days around the first treatment of mastitis. Resilience indicators based on variability in milk yield might reflect resistance to mastitis. However, METB and DIGS were negatively genetically correlated with Lnsd2, in contrast to UDDE and REPR, which was not expected. This might be a statistical artifact. In this study, METB included milk fever, ketosis, and displacement of abomasum which is mainly concentrated in early lactation, and ML and Lnsd2 are lower in early lactation than mid and late lactation. This might have caused the misleading impression that ML and Lnsd2 were less in cows which had METB. Meanwhile, the incidence of UDDE, PRER, METB, and DIGS in the population was 29.2%, 10.7%, 6.5%, and 2.0%, respectively. The imbalance in the raw data for the two binary traits (METB and DIGS) also affected the genetic correlation accuracy and was the main reason for the high SE of the genetic correlation estimates for DIGS. Poppe et al. (2020, 2021a, 2021b, 2021c) used moving average, moving median, Wilmink model, and quantile regression models on raw daily milk yield to explore and validate the variance of deviation, autocorrelation, and skewness of daily milk yield, and the results similarly demonstrated the potential of the variance as resilience indicator. A major difference between our study and theirs was how the lactation curves were fitted. A single longitudinal trait is unlikely to be sensitive to all environmental disturbances. When resilience indicators are defined using other longitudinal traits (e.g., feed intake, activity level), additional resilience mechanisms might be captured. Poppe et al. (2022a) showed that fluctuations on daily step count data are more sensitive to hoof health, fertility, and body condition score. Therefore, the use of multiple high-throughput monitoring data to study resilience in dairy cattle can avoid a heavy reliance on a single trait (milk yield) and be more useful to herds in determining and breeding more resilient cows.

There was a high positive genetic correlation between Lnsd2 and MY305 and longevity traits, indicating that more productive cows tend to be less resilient. The negative correlation between resilience and milk yield may be explained based on the “Resource Allocation Theory” (Rendel, 1963). High-producing cows tend to have fewer resources to resist environmental disturbances due to the high demand for resources for milk production. As a result, high production leads to lower resilience. Moreover, cows with high milk yield have an advantage against active culling in the herd and therefore tend to have a higher productive life (Hu et al., 2021b; Zhang et al., 2021), which might explain the lower longevity of more resilient cows. Therefore, when we improve resilience through genetic selection on resilience indicator, we should also consider milk production, the main breeding goal of dairy farming, to develop a balanced selection index for sustainable production and balanced breeding. Resilience is a comprehensive trait and its economic value is not only related to production, health, and functional traits, but also has additional economic values which are not included in the current breeding goal. For instance, high resilient cows can reduce the cost of disease treatment and human costs for herd. It would be one of the directions of our research to find evidence for Lnsd2 as a breeding target for the next generation of more resilient animals through economic analyses. In summary, the results of the genetic analyses show the high potential and merit of continuous monitoring milk records for deriving novel resilience indicators in dairy cattle breeding. Also, the genetic analyses and phenotypic validation led to the selection of Lnsd2 as the best indicator of resilience in Chinese Holstein cattle.

## 5 Conclusion

The translation of daily milk yield into fluctuations and milk loss based on ELC enables the evaluation of phenotypic and genetic responses of cows to environmental perturbations and the ability of cows to cope with perturbations. Although heritability estimates for milk loss traits are low, there is still variability which reflect variation in daily milk yield as well as the effects of environmental disturbances on cows. Log-transformed standard deviation of milk yield deviations when removing the first and last 10 DIM (Lnsd2) had the highest heritability and was favorably genetically associated with several milk loss, reproduction, longevity, and health traits, while the antagonistic relationship between resilience and milk production indicted the necessity of balanced breeding when improving resilience. In summary, Lnsd2 is recommended as the best resilience indicator among the ones evaluated in this study for genetically improving resilience in Holstein cows. This study also shows the potential of using high frequency automatic monitoring of daily milk yield to characterize and identify the milk yield dynamics during perturbations, which can be used for on-farm monitoring and precision management.

## Data Availability

The data analyzed in this study is proprietary. Requests to access the datasets used should be directed to YW: wangyachun@cau.edu.cn.
