# Supplementary material for: Exploring milk loss and variability during environmental perturbations across lactation stages as resilience indicators in Holstein cattle
Source: Front Genet. 2022 Dec 2;13:1031557. doi: 10.3389/fgene.2022.1031557 (PMC9757536; doi:10.3389/fgene.2022.1031557)
Supplement: Supplementary file 1 [file Table1.docx]

Supplementary Material

**Supplementary Table S1**| Descriptive statistics of reproduction, longevity, and health traits included in the analyses.

| Trait^1^ | N | Mean | SD | Min | Max | Coefficient of variation | |  |
| --- | --- | --- | --- | --- | --- | --- | --- | --- |
| AFC, d | 4,222 | 733.3 | 102.5 | 600 | 1,657 | | 14.0 | |
| AFS, d | 4,222 | 412.4 | 28.8 | 358 | 681 | | 7.0 | |
| IFL_H, d | 3,871 | 37.5 | 66.3 | 0 | 355 | | 176.8 | |
| IFL_C, d | 4,476 | 64.8 | 76.7 | 0 | 355 | | 118.4 | |
| ICF, d | 8,860 | 60.5 | 9.9 | 43 | 226 | | 16.4 | |
| Lon1, d | 2,610 | 371.9 | 51.4 | 241 | 1,068 | | 13.8 | |
| Lon2, d | 1,350 | 693.9 | 110.5 | 241 | 1,068 | | 15.9 | |
| PL, d | 883 | 369.6 | 157.7 | 180 | 1,068 | | 42.7 | |
| UDDE | 7,347 | 0.29 | 0.45 | 0 | 1 | | 155.2 | |
| REPR | 7,347 | 0.11 | 0.31 | 0 | 1 | | 281.8 | |
| METB | 5,921 | 0.06 | 0.25 | 0 | 1 | | 416.7 | |
| DIGS | 5,921 | 0.02 | 0.14 | 0 | 1 | | 698.3 | |

^1^N: number of records; AFC: age at first calving in heifers; AFS: age at first insemination in heifers; IFL_H: interval from first to last insemination in heifers; IFL_C: interval from first to last insemination in cows; ICF: interval from calving to first insemination; Lon1: the days from the first calving to the end of the first lactation or culling; Lon2: the days from the first calving to the end of the second lactation or culling; PL: productive life referring the days from the first calving to culling or death; UDDE: udder health; REPR: reproductive disorders; METB: metabolic disorders; DIGS: digestive disorders.

**Supplementary Table S2**| Data editing steps and numbers of cows, lactations, and records remaining after each data editing step.

|  | Item | | Condition | No. of cows | No. of lactations | No. of records |
| --- | --- | --- | --- | --- | --- | --- |
| 0 | Raw data | |  | 22,666 | 49,118 | 11,536,488 |
| 1 | Days in milk (DIM) | | 1-305 days | 21,930 | 47,161 | 9,809,348 |
| 2 | Duplicated data | | Remove | 21,930 | 47,161 | 9,801,978 |
| 3 | Daily milk yield (DMY) | | 2.5-100 kg | 21,898 | 47,066 | 9,692,500 |
| 4 | Age at first calving (AFC) | | 600-1,800 days | 21,857 | 46,994 | 9,680,818 |
| 5 | Number of records within a lactation | | > 220 | 15,018 | 27,158 | 7,631,976 |
| 6 | Time span of records within a lactation | | > 260 d | 14,283 | 25,006 | 7,118,364 |
| 7 | DIM with the first record within a lactation | | < 30 d | 14,109 | 24,586 | 7,013,361 |
| 8 | DIM with the last record within a lactation | | > 270 d | 13,930 | 23,656 | 6,772,142 |
| 9 | Consecutive days missing | | < 10 d | 13,484 | 22,366 | 6,440,601 |
| 10 | Records deviating 3 or more SD from the mean for each DIM within each category | | Remove | 13,484 | 22,366 | 6,344,000 |
| 11 | Filling in missing values using linear regression | |  | 13,484 | 22,366 | 6,821,630 |
| 12 | Fit lactation curves | Fitted value | 0-100 kg | 11,045 | 15,115 | 15,115 |
|  |  | R^2^ | > 0.75 | 9,500 | 12,586 | 12,586 |
| 13 | 305 days milk yield calculated via lactation curve deviating from 3SD from the mean | | Remove | 9,476 | 12,545 | 12,545 |
| 14 | Individuals without pedigrees (Sire and Dam were both unknown) | | Remove | 6,816 | 8,935 | 8,935 |

**Supplementary Table S3**| The silhouette coefficient of different numbers of cluster.

| No. of clusters | Silhouette coefficient |
| --- | --- |
| 4 | -0.15 ± 0.09 |
| 5 | -0.12 ± 0.11 |
| 6 | -0.11 ± 0.11 |
| 7 | -0.13 ± 0.11 |

**Supplementary Table S4**| Descriptive analyses of each lactation cluster and total dataset.

|  | Cluster | | | | | | Total |
| --- | --- | --- | --- | --- | --- | --- | --- |
|  | (a) | (b) | (c) | (d) | (e) | (f) |  |
| No. of lactation based on the dataset in Section 2.2 | 10,192 | 7,017 | 393 | 3,970 | 621 | 173 | 22,366 |
| No. of lactation based on the final dataset | 3,877 | 2,871 | 44 | 1,855 | 272 | 16 | 8,935 |
| Parity^1^ | 2.67  (1.11) | 2.12  (1.17) | 2.03  (1.18) | 1.37  (0.85) | 1.36  (0.83) | 2.12  (1.18) | 2.21  (1.05) |
| Peak yield, kg | 48.27  (10.40) | 43.20  (11.03) | 47.66  (10.89) | 39.42  (7.73) | 37.47  (6.94) | 47.52  (10.49) | 44.46  (10.68) |
| Peak day, d | 60.26  (27.43) | 84.92  (38.27) | 63.73  (38.14) | 126.78  (55.79) | 172.14  (57.40) | 84.50  (49.90) | 85.46  (49.15) |

^1^Parity, peak yield, and peak day were calculated based on the final dataset (8,935 lactations).

**Supplementary Table S5**| Distribution of four lactation curve models of the final dataset.

| Model | No. of lactations | R^2^ | Percentage of the first parity, % | No. of data per lactation after iteration |
| --- | --- | --- | --- | --- |
| Wood model | 2,042 | 0.87 (0.06) | 66.94 | 283.31 (14.23) |
| Nelder model | 791 | 0.87 (0.06) | 82.17 | 285.45 (13.87) |
| Wilmink model | 931 | 0.88 (0.06) | 31.15 | 287.33 (13.43) |
| Ali-Schaeffer model | 5,137 | 0.90 (0.06) | 37.03 | 281.75 (15.13) |
| Total | 8,935 | 0.89 (0.06) | 47.25 | 283.02 (14.76) |

**Supplementary Table S6**| The effect of cluster group and lactation curve model on ML and Lnsd2.

| Fixed effect | N | ML | Lnsd2 |
| --- | --- | --- | --- |
|  |  | LSM ± SE | LSM ± SE |
| Cluster group |  |  |  |
| (a) | 3,877 | 309.75 ± 5.91^e^ | 1.14 ± 0.01^e^ |
| (b) | 2,871 | 324.06 ± 6.09^d^ | 1.20 ± 0.01^d^ |
| (c) | 44 | 678.55 ± 25.92^a^ | 1.76 ± 0.05^a^ |
| (d) | 1,855 | 346.91 ± 7.10^c^ | 1.26 ± 0.01^c^ |
| (e) | 272 | 399.89 ± 11.91^b^ | 1.39 ± 0.02^b^ |
| (f) | 16 | 788.42 ± 42.44^a^ | 1.92 ± 0.08^a^ |
| Lactation curve model |  |  |  |
| Wood model | 2,042 | 484.01 ± 10.39^b^ | 1.45 ± 0.02^b^ |
| Nelder model | 791 | 459.79 ± 11.41^c^ | 1.41 ± 0.02^c^ |
| Wilmink model | 931 | 454.61 ± 11.14^c^ | 1.39 ± 0.02^c^ |
| Ali-Schaeffer model | 5,137 | 499.99 ± 9.87^a^ | 1.50 ± 0.02^a^ |

^a-e^ Different letter superscripts among different levels mean significant difference (*P* < 0.05), and the same letter superscripts mean no significant difference (*P* > 0.05). ML: sum of the milk yield which dropped in all fluctuation phases in a lactation; Lnsd2: log-transformed standard deviation of milk deviations based on the lactation when removing first and last 10 DIM.

**Supplementary Table S7**| Comparison of top 20% and bottom 20% estimated breeding values (EBVs) in the validation dataset of Lnsd1.

| Trait^1^ | N | Top 20% | Bottom 20% | *P* value |
| --- | --- | --- | --- | --- |
| EBV | 116 | -0.02±0.01 | 0.01±0.01 | < 0.01** |
| Lnsd1 | 116 | 0.92±0.35 | 0.93±0.42 | 0.40 |
| MY305, kg | 116 | 8,861.19±1,879.18 | 8,006.07±1,891.60 | < 0.01** |
| ML, kg | 116 | 206.38±137.35 | 235.70±176.28 | 0.16 |
| NML, time | 116 | 3.69±1.41 | 3.84±1.46 | 0.22 |
| TDML, d | 116 | 70.11±27.72 | 73.48±29.06 | 0.37 |
| MLP, % | 116 | 2.47±2.48 | 3.22±2.81 | 0.02* |
| AFC, d | 116 | 700.43±49.30 | 711.98±76.06 | 0.09 |
| AFS, d | 116 | 416.55±9.06 | 405.80±22.43 | < 0.01** |
| IFL_H, d | 108 | 12.51±30.77 | 25.78±45.03 | 0.01* |
| ICF, d | 113 | 66.27±7.29 | 64.11±5.94 | 0.02* |
| Lon1, d | 59 | 380.10±67.18 | 371.56±43.29 | 0.21 |
| Lon2, d | 19 | 685.26±105.04 | 634.89±159.71 | 0.13 |
| UDDE | 98 | 0.26±0.44 | 0.21±0.41 | 0.50 |
| REPR | 98 | 0.27±0.44 | 0.22±0.42 | 0.51 |
| METB | 109 | 0.03±0.16 | 0.05±0.21 | 0.24 |
| DIGS | 109 | 0.05±0.21 | 0.04±0.19 | 0.63 |

^1^EBV: estimated breeding value; Lnsd1: log-transformed standard deviation of deviations based on the entire lactation; MY305: 305 days milk yield; ML: sum of the milk yield which dropped in all fluctuation phases in a lactation; NML: number of milk loss events; TDML: total number of days for milk loss per lactation; MLP: the percentage of ML to MY305; AFC: age at first calving in heifers; AFS: age at first insemination in heifers; IFL_H: interval from first to last insemination in heifers; ICF: interval from calving to first insemination; Lon1: the days from the first calving to the end of the first lactation or culling; Lon2: the days from the first calving to the end of the second lactation or culling; UDDE: udder health; REPR: reproductive disorders; METB: metabolic disorders; DIGS: digestive disorders.

**Supplementary Table S8**| Comparison of top 20% and bottom 20% estimated breeding values (EBVs) in the validation dataset of Lnsd3.

| Trait^1^ | N | Top 20% | Bottom 20% | *P* value |
| --- | --- | --- | --- | --- |
| EBV | 128 | -0.01±0.02 | 0.06±0.01 | < 0.01* |
| Lnsd3 | 128 | 0.76±0.35 | 0.82±0.35 | 0.07 |
| MY305, kg | 128 | 8,702.12±1,808.22 | 8,645.40±2,251.59 | 0.41 |
| ML, kg | 128 | 204.15±152.92 | 212.80±157.12 | 0.66 |
| NML, time | 128 | 3.59±1.35 | 3.60±1.39 | 0.46 |
| TDML, d | 128 | 68.66±26.39 | 67.54±28.74 | 0.74 |
| MLP, % | 128 | 2.43±2.01 | 2.78±2.60 | 0.11 |
| AFC, d | 128 | 703.83±52.60 | 708.31±73.31 | 0.29 |
| AFS, d | 128 | 415.16±11.59 | 406.95±21.76 | < 0.01** |
| IFL_H, d | 120 | 17.45±39.82 | 21.61±39.05 | 0.41 |
| ICF, d | 126 | 65.83±6.64 | 65.86±7.24 | 0.98 |
| Lon1, d | 65 | 384.43±72.96 | 366.05±5.98 | 0.02* |
| Lon2, d | 22 | 666.77±113.48 | 638.32±145.42 | 0.24 |
| UDDE | 105 | 0.31±0.47 | 0.29±0.45 | 0.65 |
| REPR | 105 | 0.26±0.44 | 0.15±0.36 | 0.06 |
| METB | 119 | 0.03±0.16 | 0.03±0.18 | 0.35 |
| DIGS | 119 | 0.04±0.19 | 0.01±0.07 | 0.98 |

^1^EBV: estimated breeding value; Lnsd3: log-transformed standard deviation of deviations during the lactation peak period; MY305: 305 days milk yield; ML: sum of the milk yield which dropped in all fluctuation phases in a lactation; NML: number of milk loss events; TDML: total number of days for milk loss per lactation; MLP: the percentage of ML to MY305; AFC: age at first calving in heifers; AFS: age at first insemination in heifers; IFL_H: interval from first to last insemination in heifers; ICF: interval from calving to first insemination; Lon1: the days from the first calving to the end of the first lactation or culling; Lon2: the days from the first calving to the end of the second lactation or culling; UDDE: udder health; REPR: reproductive disorders; METB: metabolic disorders; DIGS: digestive disorders.

**Supplementary Table S9**| Comparison of top 20% and bottom 20% estimated breeding values (EBVs) in the validation dataset of Lnsd4.

| Trait^1^ | N | Top 20% | Bottom 20% | *P* value |
| --- | --- | --- | --- | --- |
| EBV | 125 | -0.01±0.01 | 0.04±0.01 | < 0.01** |
| Lnsd4 | 125 | 0.60±0.39 | 0.61±0.41 | 0.49 |
| MY305, kg | 125 | 8,750.93±1,817.16 | 8,513.79±2,080.25 | 0.17 |
| ML, kg | 125 | 190.78±124.98 | 203.34±147.04 | 0.47 |
| NML, time | 125 | 3.51±1.34 | 3.61±1.42 | 0.29 |
| TDML, d | 125 | 68.20±27.53 | 68.98±27.93 | 0.82 |
| MLP, % | 125 | 2.24±1.56 | 2.69±2.46 | 0.08 |
| AFC, d | 125 | 702.03±50.08 | 711.45±79.73 | 0.13 |
| AFS, d | 125 | 415.03±12.19 | 400.31±23.30 | < 0.01** |
| IFL_H, d | 117 | 14.93±35.30 | 30.94±54.33 | 0.01* |
| ICF, d | 123 | 66.31±7.06 | 64.63±5.94 | 0.05* |
| Lon1, d | 64 | 380.47±65.06 | 363.98±9.75 | 0.05* |
| Lon2, d | 21 | 667.43±116.24 | 690.76±124.09 | 0.73 |
| UDDE | 104 | 0.32±0.47 | 0.28±0.45 | 0.55 |
| REPR | 104 | 0.24±0.43 | 0.16±0.37 | 0.17 |
| METB | 117 | 0.03±0.18 | 0.04±0.20 | 0.37 |
| DIGS | 117 | 0.03±0.18 | 0.01±0.09 | 0.18 |

^1^EBV: estimated breeding value; Lnsd4: log-transformed standard deviation of milk deviations based on the period consisting of each DIM when the actual milk yield was below the expected lactation curve (ELC) fitted value; MY305: 305 days milk yield; ML: sum of the milk yield which dropped in all fluctuation phases in a lactation; NML: number of milk loss events; TDML: total number of days for milk loss per lactation; MLP: the percentage of ML to MY305; AFC: age at first calving in heifers; AFS: age at first insemination in heifers; IFL_H: interval from first to last insemination in heifers; ICF: interval from calving to first insemination; Lon1: the days from the first calving to the end of the first lactation or culling; Lon2: the days from the first calving to the end of the second lactation or culling; UDDE: udder health; REPR: reproductive disorders; METB: metabolic disorders; DIGS: digestive disorders.
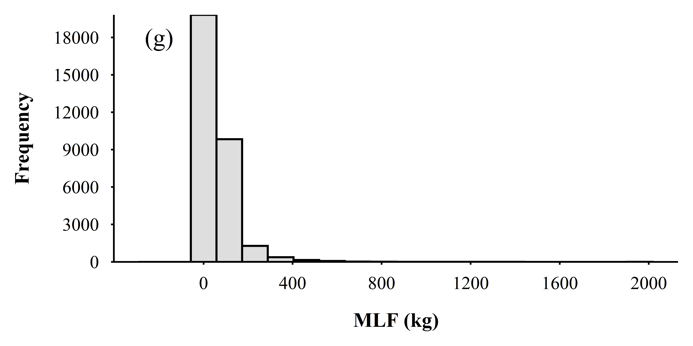

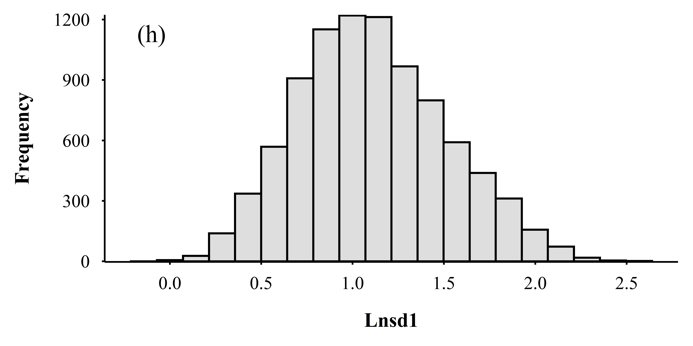

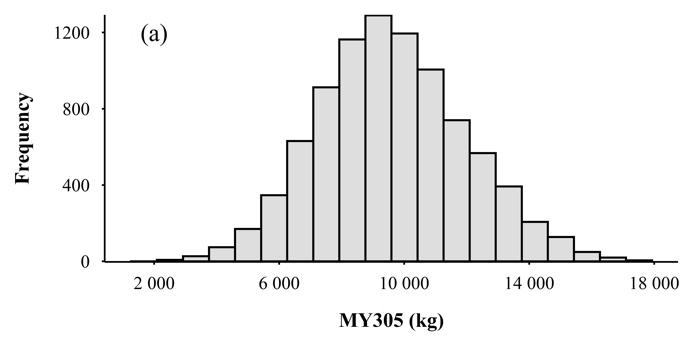

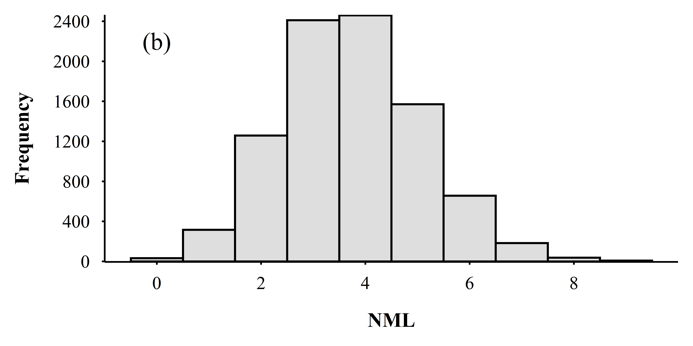

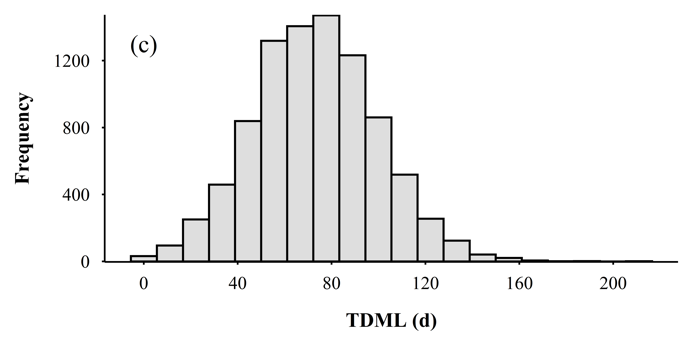

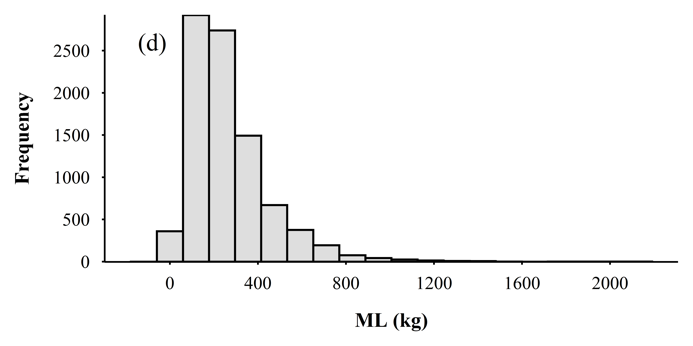

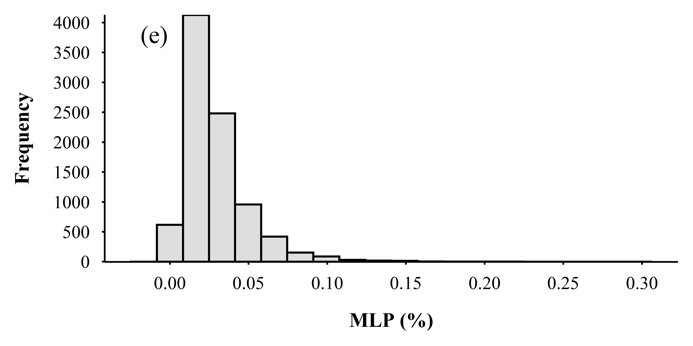

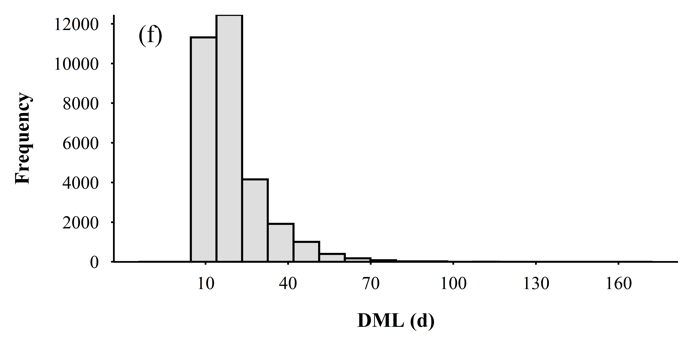


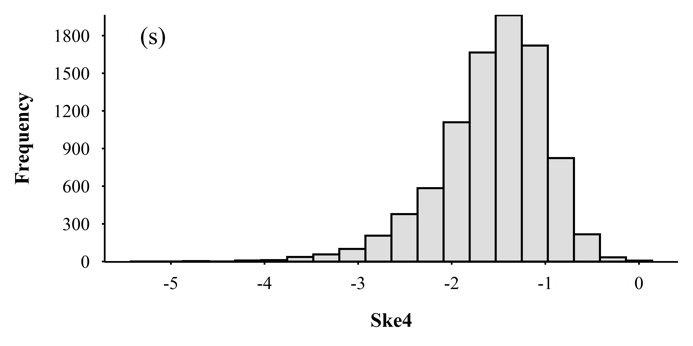

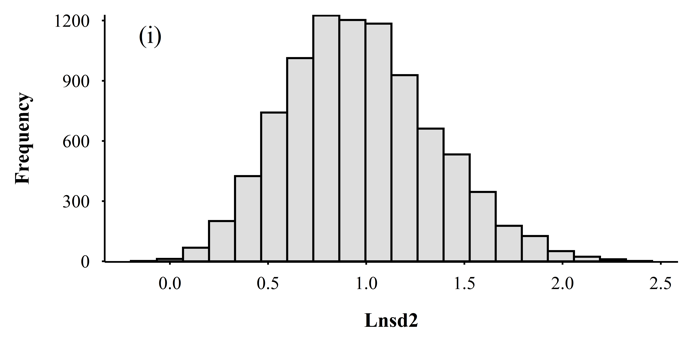

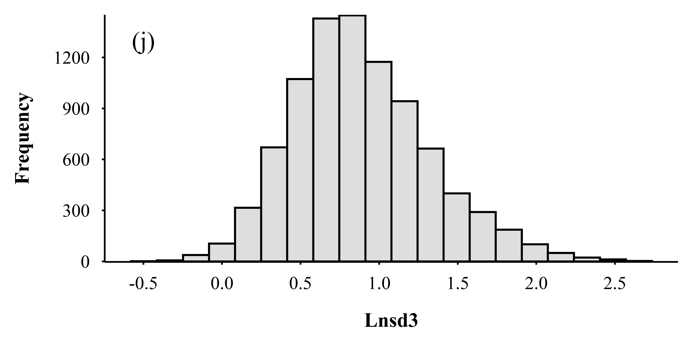

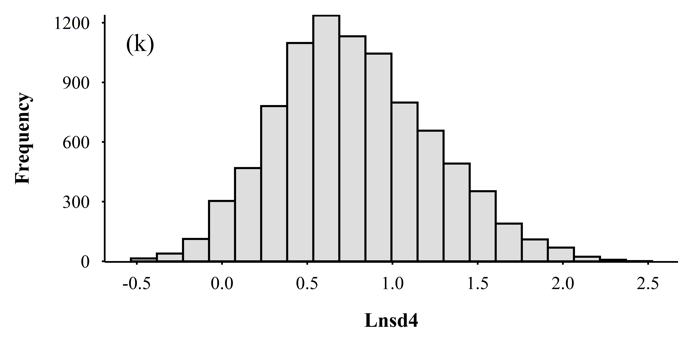

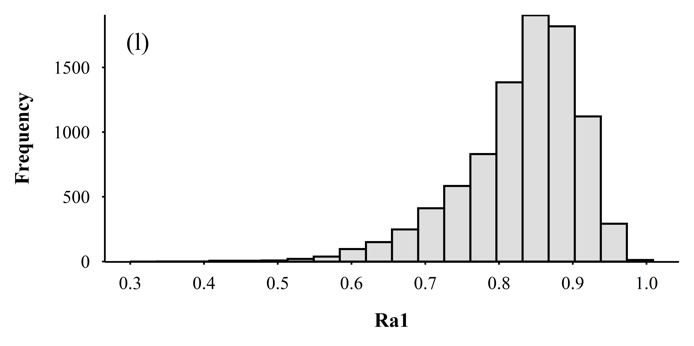

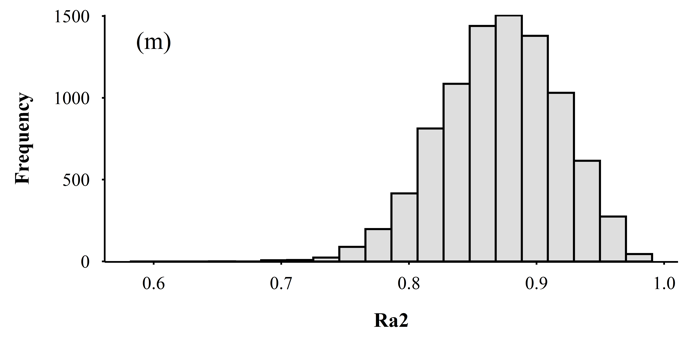

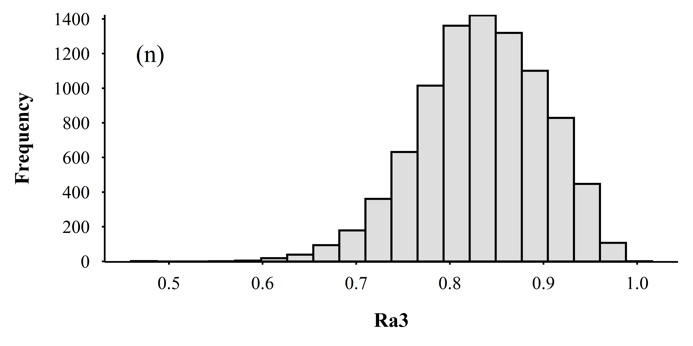

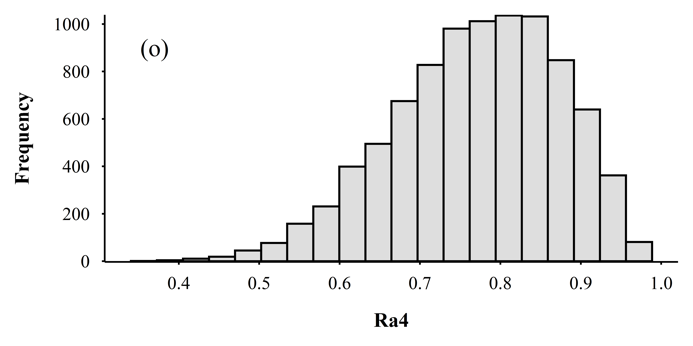

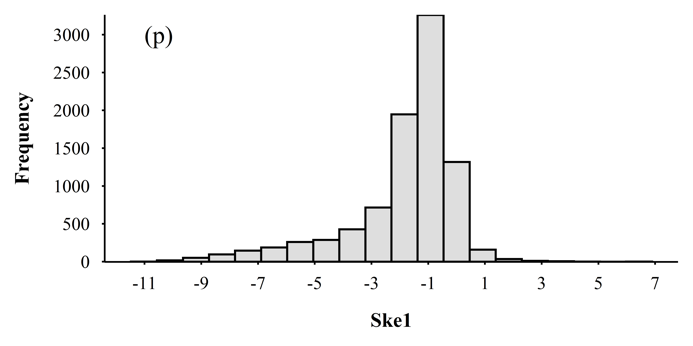

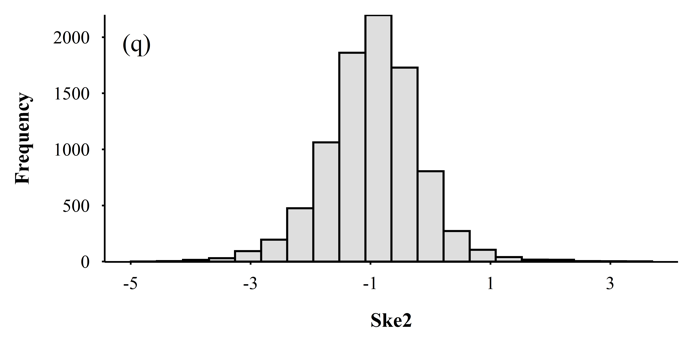

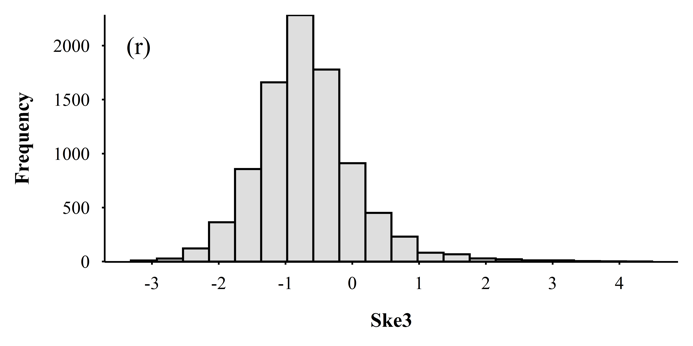


**Supplementary Figure S1**| The distributions of MY305 and resilience indicators. MY305: 305 days milk yield; NML: number of milk loss events; TDML: total number of days for milk loss per lactation; ML: sum of the milk yield which dropped in all fluctuation phases in a lactation; MLP: the percentage of ML to MY305; DML: length of each milk loss period in days; MLF: milk loss in each milk loss period; Lnsd: log-transformed standard deviation of milk deviations; Ra: lag-1 autocorrelation of milk deviations; Ske: skewness of milk deviations. These three variability traits were calculated based on records from the entire lactation (Lnsd1, Ra1, and Ske1, from DIM 1-305), lactation period when removing the first and last 10 days (Lnsd2, Ra2, and Ske2, from DIM 11-295), during the lactation peak period (Lnsd3, Ra3, and DIM 60-90), and the period consisting of each DIM when the actual milk yield was below the expected lactation curve (ELC) fitted value (Lnsd4, Ra4, and Ske4), respectively.
